# Indigenous uses of wild and tended plant biodiversity maintain ecosystem services in agricultural landscapes of the Terai Plains of Nepal

**DOI:** 10.1186/s13002-020-00382-4

**Published:** 2020-06-08

**Authors:** Jessica P. R. Thorn, Thomas F. Thornton, Ariella Helfgott, Kathy J. Willis

**Affiliations:** 1 Department of Zoology, Biodiversity Institute, University of Oxford, New Radcliffe House, Radcliffe Observatory Quarter, Woodstock Road, Oxford, OX2 6GG UK; 2 Department of Environment and Geography, York Institute of Tropical Ecology, University of York, 290 Wentworth Way, Heslington, York, YO1 5NG UK; 3 African Climate and Development Initiative, University of Cape Town, Upper Campus, Geological Sciences Building Level 6, 13 Library Road, Rondebosch, 7700 South Africa; 4 Environmental Change Institute, Oxford University Centre for the Environment, University of Oxford, S Parks Rd, Oxford, OX1 3QY UK; 5 School of Arts and Sciences, University of Alaska Southeast, Juneau, AK 99801 USA; 6 Kew Royal Botanical Gardens, Richmond, TW9 3AB UK; 7 Department of Biology, University of Bergen, Postboks 7803, 5020 Bergen, Norway

**Keywords:** Agrobiodiversity conservation, Ethnopharmacology, Ethnobotany, Ethnoecology, Ethnomedicine, Food security, Indigenous knowledge, Medicinal plants, Traditional Ecological knowledge

## Abstract

**Background:**

Despite a rapidly accumulating evidence base quantifying ecosystem services, the role of biodiversity in the maintenance of ecosystem services in shared human-nature environments is still understudied, as is how indigenous and agriculturally dependent communities perceive, use, and manage biodiversity. The present study aims to document traditional ethnobotanical knowledge of the ecosystem service benefits derived from wild and tended plants in rice-cultivated agroecosystems, compare this to botanical surveys, and analyze the extent to which ecosystem services contribute social-ecological resilience in the Terai Plains of Nepal.

**Method:**

Sampling was carried out in four landscapes, 22 Village District Committees, and 40 wards in the monsoon season. Data collection was based on transects walks to collect plant specimens, structured and semi-structured interviews, and participatory fieldwork in and around home gardens, farms, and production landscapes. We asked 180 farmers to free-list vernacular names and describe use-value of wild and tended plants in rice-cultivated agroecosystems. Uses were categorized into eight broad groupings, and 61 biomedical ailment classifications. We assessed if knowledge of plant species diversity and abundance differed with regard to caste, age, and gender.

**Results:**

Nepalese farmers have a deep knowledge of the use and management of the 391 vascular plant specimens identified, which provide key provisioning, regulating, supporting, and cultural ecosystem services. Altogether, plants belong to 76 distinct plant species from 49 phylogenetic families: 56 are used to cure 61 ailments, 27 for rituals, 25 for food, 20 for timber, 17 for fuel, 17 for fodder, 11 for soil enhancement, and eight for pesticides. Four caste groups have statistically different knowledge, and younger informants report a lower average number of useful plants.

**Conclusion:**

Agricultural landscapes in Nepal are reservoirs of biodiversity. The knowledge of the use of wild and tended plant species in and around these farms differs by the caste and age group of land manager. Conducting research on agroecosystems will contribute to a deeper understanding of how nature is perceived by locals, to more efficient management and conservation of the breadbasket of Nepal, and to the conservation of valuable, but disappearing traditional knowledge and practice.

## Background

As the costs of agricultural expansion and land conversion begin to accumulate (e.g., habitat destruction and fragmentation, changes in hydrological and biogeochemical cycles, land use change emissions [1]), and other pressures on biodiversity escalate (e.g., overharvesting, invasive species, increased extinction rates), a realization in the fields of conservation and agricultural sciences has developed regarding the indispensable role that biodiversity plays in food security and the wider provisioning of crucial goods and services [2, 3]. This is particularly the case for the livelihoods of many indigenous communities that have limited access to external production inputs [4, 5]. Moreover, in a world with a global population growing toward 10 billion people, land is a fixed resource, and land use change today has implications for the services it can provide in subsequent decades [6]. Increased evidence shows that continued ecosystem degradation will likely lead to negative feedbacks that reduce agricultural yields and increase the likelihood of abrupt system change [7]. Yet, in practice, this recognition has consistently been ignored [8]. In light of these challenges, the Sustainable Development Goals (SDGs) call for a comprehensive new approach to “ensure sustainable consumption and production patterns” [9] and “protect, restore, and promote the sustainable use of terrestrial ecosystems” [5, 10]. Although dietary change [9, 11, 12], increased investment, policy reform [13], biotechnology, and many other proposed solutions hold promise, understanding changing local ethnobotanical knowledge, and how communities facilitate ecosystem service delivery can substantially help turn farmer’s skills at biomanipulation to work for biodiversity conservation, and appropriately account for multiplicity of values inherent in diverse, cultivated landscapes [14–16].

A growing body of literature suggests small- to medium-sized farming communities in remote, marginal areas may support among the highest overall biodiversity levels of any agricultural system [4]. For example, farmers maintain multiple layered systems of trees, herbs, climbers, grasses, and herbs in and around their farms [17, 18]. These farming systems typically have higher variation in plant community abundance compared to monoculture croplands [19] and include wild relative species often considered more resilient than modern cultivars [20, 21]. Moreover, landscapes are often configured as multifunctional and relational mosaics, with crops situated according to both their utility and complementarity with other biota [22–24]. Such landscape heterogeneity and connectivity between croplands and native vegetation can encourage the recolonization of disturbed habitats, and counterbalance degraded ecosystem function [25, 26]. Understanding local agricultural traditions and preferences for wild and tended plant species^1^ is important because processes of planting, extraction, and domestication of plant populations influence the community structure, rate of species turnover [18], genetic makeup [27], as well as local responses to environmental change and degradation [28, 29]. This is particularly the case in Nepal, which has a long historical tradition of farming, with an estimated 78% of the current population actively engaged in the management of agro-biodiverse landscapes. Indeed, the region has been a rich source of valuable plant species since Vedic periods (3400–1600 BC) [30]. Even today, many small to medium farmers still continue to rely heavily on provisioning ecosystem services provided by plants around farms for their basic necessities [31, 32]. Meanwhile, indigenous knowledge of plant utilization is rapidly changing, with the increased availability of substitutes, synthetic inputs, changing vocations and more affluent lifestyles [30].

Despite a rapidly accumulating evidence base quantifying ecosystem services, uncertainties remain about the role of biodiversity in the maintenance of ecosystem services in variable human-dominated landscapes, humans contributions to biodiversity maintenance, and the extent to which such services contribute to social-ecological resilience [25, 26, 28, 29]. Furthermore, precise information of local ethnobotanical knowledge, including how communities facilitate ecosystem service delivery at the landscape and community level to benefit from genetic resources remains limited [8, 12, 25, 26]. In Nepal, most studies have focused only on ecological structure or specific ecosystem services [33], while ethnobotanical studies have tended to focus on plants with pharmacological value [34–36] or cultural keystone species [37], neglecting a more comprehensive assessment of ecosystem goods and services values at the landscape scale. In the Terai, although a few scholars have studied the traditional knowledge systems of the Tharu [38], many regions remain understudied. No studies have considered how small- to medium-sized farmers mediate non-agricultural wild and tended plant species community composition in and around farms.

Here, we explore the role of biodiversity in maintaining ecosystem services in the Terai Plains of Nepal by investigating the composition and use of wild and tended plant material found in and around rice production landscapes. Specifically, the paper is founded on the following objectives: first, to survey wild and tended plant abundance and diversity; second, to capture local ethnobotanical knowledge including use, source, and administration of plants and determine if knowledge of use differs according to caste, gender, and age; and third, identify what factors incentivize the maintenance of biodiversity in and around farms.

## Materials and methods

### Study area

Study sites spanned the Central and Western zones of the Terai Plains (hereafter referred to as the Terai), a unique physio-geographic zone along the foothill of the Himalayas across the South of Nepal, stretching 1360 km East-West and 25–32 km North-South. Elevation ranges from 108 to 658 masl (a 550 masl range) and soils are laterite. The climate is the warm-temperate Indo-Malayan Tropical Monsoon zone, with a mean annual temperature of 24.6 °C (min = 18.2 °C, max = 31 °C), while rainfall ranges from 1000 to 2100 mm/year. Despite its relatively small area, the area provides a unique assemblage of very different habitats and holds high levels of biodiversity [39]. Similarly, the Terai accounts for 68% of the country’s agricultural output [40], 43% of total cultivated land, and 21% of land cover and supports much of the country’s population [28]. This is largely due to fertile soils from flat alluvial deposits and has led to the region being described as the “food basket” or “granary” [29, 40] of the country. Yet, production does not meet the demands of the population, with only 16% of the land under arable cultivation [41], contributing to food insecurity and malnutrition rates of ~ 43% [42]. Concurrently, inhabitants are prone to frequent risks, such as flooding, sediment accumulation, and inundation over large stretches of land adjoining banks of rivers which debouch in the Terai from higher Himalayas [29].

The region is also home to the Tharu, which represent the largest ethnic indigenous minority of Nepal—comprising of over 2000 subdivisions. Historically, the Tharu were semi-nomadic, practicing short fallow shifting rice cultivation with livestock; however, today many are increasingly sedentary. Most people farm rice (*Oryza sativa L.*), lentils (*Lens culinaris* Medic), maize (*Zea mays* L.), wheat (*Triticum aestivum*), and mustard (*Brassica juncea*) using rainfed irrigation (73.5%) while some are also laborers, community workers, or small business owners but still keep livestock at home) as part of their livelihood strategy [29]. Few public health dispensaries provide basic facilities but people living in more remote locations have limited access to them. They mainly depend on herbal remedies prepared at home or by traditional healers.

As is common in many rural agroecosystems across the globe [43], significant change has occurred in the Terai in the last 50 years: with the advent of Dichlorodiphenyltrichloroethane, the eradication of malaria and 1964 Land Act, where land was made freely available for people from the Mid-Hills region. As a result, rice production increased with concomitant deforestation and biodiversity loss [44]. During the resettlement programme, the cultural-demographic profile shifted from small pockets of Tharu to a mixture of Brahmin, Chettri, other castes, and Indian migrants [45]. This amalgamation, in turn, has led to homogenization of culture and knowledge, resulting in a loss of ethnobotanical knowledge, identities, and agricultural practices which sustain Tharu livelihoods. Changing land rights, urbanization, and new income streams have also contributed to these shifts [38].

### Sampling and plant identification

**Fig. 1:**
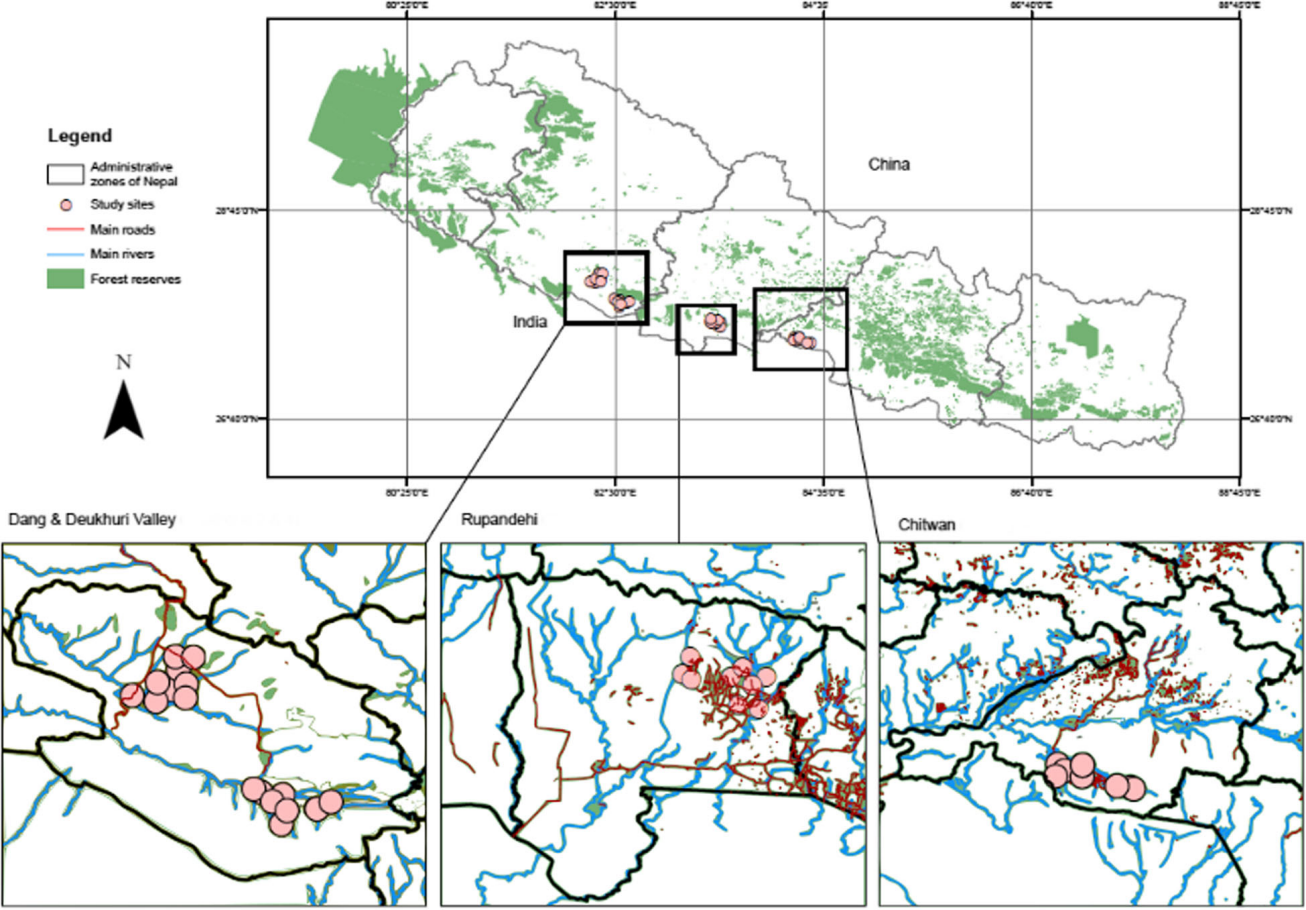
Map of study area in the Central and Western zones of the Terai Plains of Nepal (*n* = 40 villages)

Field sampling was conducted during the monsoon season (May–September) in 2012-2014. Sampling was carried out in four landscapes, 22 VDCs, and 40 wards (Fig. 1): (1) four village district committees (VDCs) in Madi Valley, Chitwan district (N27° 28.305′ E084° 17.244′, 204 masl), (2) six VDCs in Rupandehi district (N27° 35.414′ E083° 31.180′, 138 masl), (3) six VDCs surrounding Gohari, Dang district (N27° 50.783′ E082° 30.068′, 256 masl) (referred to hereafter as Dang), and (4) six VDCs in the Deukhuri Valley, Dang district (N28° 03.086′ E082° 18.712′, 597 masl) (Deukhuri). Standardized sampling procedures were used to collect specimens [46] involving transects walks in home gardens, farms, and the surrounding landscape within 250 m of homesteads (one sample/species/farm). To identify Scientific and English names of species, the nomenclature followed was that employed by Press et al. (2000) [47]. To verify uses, we consulted previous studies. We were limited to collecting predefined “key” parts of the plant (e.g., leaf, fruit, stem), rather than the entire plant. Yield was not recorded. All biological material was photographed for further reference. For the purpose of this paper, biological material’ refers to wild and tended plants found in and around farms within rice-cultivated agroecosystems. They are natural materials that comprise part of or a whole living organism. Unidentified species were identified and deposited in the National Herbarium and Plant Laboratories Godawari, Lalitpur in Kathmandu.

A “site” is a rice farm (5.78 ± 2.33 ha) where terraced rice is cultivated during the monsoon season, when 80% of rain falls. In each region, ten farms were surveyed across the hydro shed catchment within 200 km^2^ using regional topographical maps of Nepal (1:25,000), sourced in 2012 from East View Cartographic Inc., Minneapolis in the USA, and the Ministry of Land Reform and Survey, Government of Nepal in Kathmandu [29].

### Ethnobotanical data collection

Ethnobotanical data were gathered from 180 informants using semi-structured interviews, questionnaire, focus group discussion, rapid participant observation, and field observation. As far as possible, the sample was randomly stratified across age (25–67 years), gender (72.5% male, 27.5% female), and caste (*n* = 10). Further, 82.5% of the study population own land through inheritance, 12.5% through procurement, and 5% through government provision [29]. Farmers (*n* = 40) managing the farms surveyed were asked to free-list vernacular names in Nepali (N) or Tharu (Th), rather than using predefined categories to reduce researcher bias [48], and describe use-value of biological material (e.g., medicine for humans/livestock, fodder, fuel, building material, biocides, food additives, fertilizers, or cultural, religious, esthetic, ornamental, and ritual purposes). Community members, rather than specialist practitioners, were interviewed to assess widely available knowledge [49]. Uses were then categorized into eight broad uses, and for plants with medicinal value into 61 ailment groupings using biomedical terminology. For each species, informants were asked to identify the part of the plant used (e.g., bark, root), the plant’s source (e.g., hedgerow, forest, riparian buffer zone), preparation and administration (e.g., decoction), timing of harvest (e.g., season), and growth form of the plant (e.g., grass, tree, shrub). Multipurpose tree species found on farms and around homesteads were recorded through visual observations. Data inconsistencies were verified through focus groups and semi-structured interviews (*n* = 140). In addition, participant observation involved observation of cultivation techniques, ritual celebrations, daily worship, indigenous folklore expressing societal cultural ties to the crop, and food traditions including preparation and occasions of consumption [50]. The results presented in this study are derived from these surveys and comprise original data.

### Statistical analysis

Plant diversity was calculated using the Shannon–Weaver diversity index: a measure of biodiversity which accounts for species dominance (richness and proportion of each species) within the community, in which *s* is the number of individuals and *p*_*i*_ is the relative proportion of individuals belonging total (*i*) individuals [51].

1 $$ \mathsf{Shannon}\hbox{-} \mathsf{Weaver}:\mathsf{H}\hbox{'}=\hbox{-} \sum \limits_{\mathsf{i}=\mathsf{1}}^{\mathsf{S}}\left({\mathsf{p}}_{\mathsf{i}}\ast \mathsf{In}\;{\mathsf{p}}_{\mathsf{i}}\right) $$

Across all and each region, one-way analysis of variance (ANOVA) compared means of diversity and absolute abundance and Pearson’s chi-squared goodness of fit tests compared proportional plant abundance, as well as knowledge of diversity and abundance with regard to caste, age, and gender. Multivariate statistics were used to assess the relationship between plant abundance and caste, using hierarchical cluster analysis using the group average, and the corresponding SIMPROF test for non-metric multidimensional scaling. Shapiro-Wilk tests were performed to assess whether data met assumptions of normality and were log/log10 transformed where necessary. Unique species found in each region were then tabulated. Data were analyzed in RStudio V.3.1.1 [52]., using the Lattice package [30] and Primer-E [53].

## Results

### Plant diversity and abundance

Overall, 391 vascular plant specimens were collected and identified as belonging to 75 distinct plant species from 49 phylogenetic families. Individual farms have between one and 27 useful (i.e., important and commonly used) plants. Across the study area, species diversity (*H*′) is 3.09 ± 0.09 (mean ± SE), species abundance is 9.75 ± 0.74, and the average number of useful plant species/farm is 9.75 ± 4.71 (Table 1).

**Table 1:** Relative diversity, abundance, and use categories of plant species

	Chitwan		Rupandehi		Deukhuri		Dang		All farms
Species diversity	3 ± 0.26		3.22 ± 0.17		3.14 ± 0.14		2.99 ± 0.08		3.09 ± 0.08
Species abundance	9.4 ± 1.66		12.1 ± 2.06		9.6 ± 1.06		7.9 ± 0.69		9.75 ± 0.74
	Total sp.	Unique sp.	Total sp.	Unique sp.	Total sp.	Unique sp.	Total sp.	Unique sp.	All sp.
All uses	38	5	49	11	44	6	31	5	75
Fuel	11	1	10	0	12	1	15	3	17
Fodder	8	2	9	1	11	2	8	2	17
Food	11	2	15	2	13	2	12	3	25
Timber	15	3	11	1	14	2	13	1	20
Soil	8	1	6	0	9	2	4	0	11
Medicine	29	2	38	8	37	4	24	4	56
Spiritual	15	2	21	5	12	2	8	1	27
Pesticide	4	0	8	1	6	0	4	0	8

### Plant utilization

**Fig. 2:**
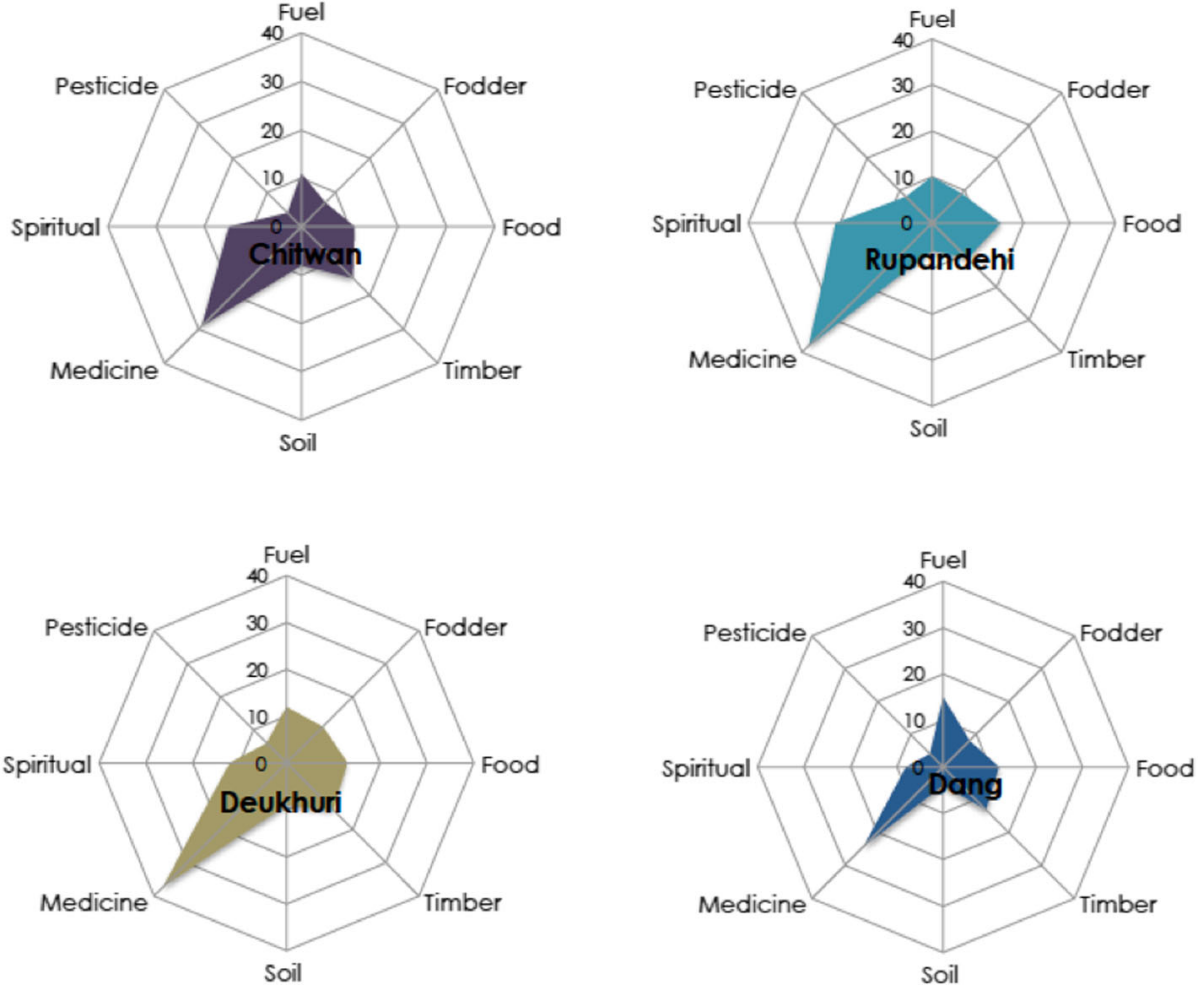
Multiple uses of wild and tended plants across four landscapes in the Terai Plains of Nepal

Of the 75 plant species collected, 56 are used for medicine, 27 for spiritual or ritual purposes, 25 for food, 20 for timber, 17 for fuel, 16 for fodder, 11 for soil enhancement, and eight for pesticides. Most plants (73.3%) have multiple uses: between two (29.3%) up to six (2.7%) purposes. Eight species are considered to have disservices (e.g., invasive weeds). The most dominant plant families are *Euphorbiaceae* (five families), *Fabaceae* (four), *Moraceae* (four), *Anacardiaceae, Lamiaceae*, and *Rutaceae* (three). The most common species are *Shorea robusta* (Sal tree) (6.7%), *Dalbergia sisoo* (Indian rosewood) (6.4%), *Azadirachta indica* (Mugwort) (6.4%), *Melia azedarach* (Persian lilac) (5.1%), *Leucaena leucocephala* (Leucaena) (4.4%), *Ficus religiosa* (Banaya tree) (4.4%), *Dendrocalamus strictus* (Bamboo) (4.4%), *Ocimum tenuiflorum* (Holy basil) (4.4%), *Mangifera indica* (Mango) (3.9%), and *Jatropha curcas* (Physic nut) (3.1%) [54–56]. Figure 2 illustrates how use differed across the landscapes sampled.

The table presents the results of our analysis of (i) species diversity; (ii) absolute abundance; and (iii) the relative constitution of number of plant species, according to use categories. Unique plant species are found in each of region, and the highest number is found in Rupandehi (values show the mean ± SE; sp.: species).

The figure shows medicinal use emerged as the dominant use category across all landscapes. Numbers refer to percentage.

### Source of biological material

**Fig. 3:**
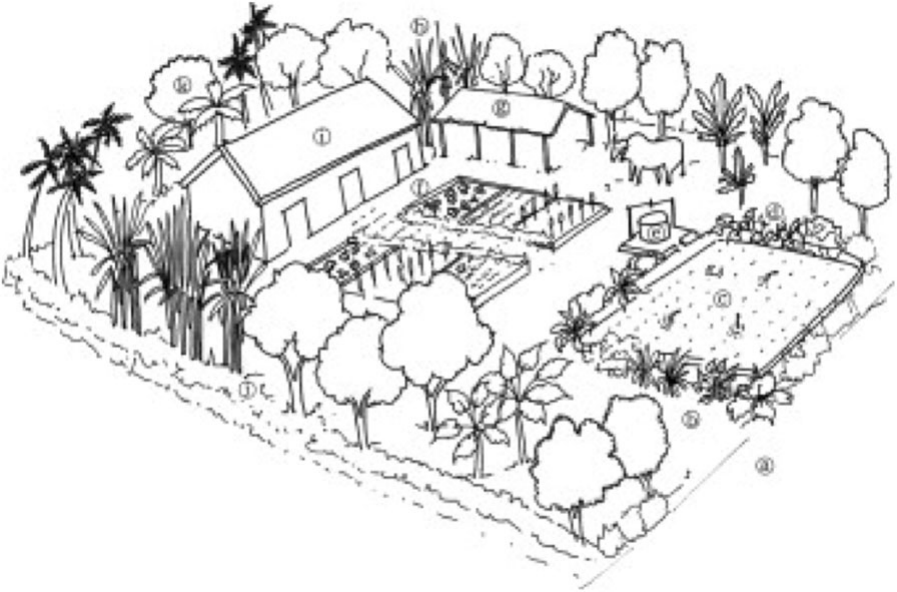
Composite schematic view illustrating a typical multi-layered, heterogeneous, integrated cropping system in the Terai. **a** road; **b** pathway; **c** rice paddy fields; **d** lentil and soya bean grown along boundaries; **e** tube wells or slurry processing for biogas; **f** vegetable garden (e.g., bottle gourd, cucumber, tomato, beans, okra, sesame), spices (e.g., ginger, turmeric), and cosmetics (e.g., aloe vera) with mulched patches and ridges/bunds for water efficiency; **g** buffalo, goat, or pig pen and fuel wood storage; **h** cluster of trees alongside boundary for windbreaks (e.g., *Dendro clamus strictus*), fuel wood and timber (e.g., *Dalbergia sisoo*, *Shorea robusta*, *Melia azedarach*), fruit (e.g., *Psidium guajava*), fodder (e.g., *Azadirachta indica*, *Abizia lebbeck*), religious value (e.g., *Aegle marmelos*), or shade (e.g., *Magnifera indica*); **i** house roof made of reed thatch, covered with creepers and gourds for esthetic value, insulation, temperature regulation, and food (Adapted from [33])

Biological material is collected from farm boundaries, around homesteads, home gardens, or uncultivated patches, such as wetlands, small woodlands, or riverbanks. Farmed areas are typically adjacent to homesteads, around which farmers maintain wind and shade barriers, nurseries, fruit orchards, ornamentals, spices, vegetables, multi-storied crops (e.g., grasses, herbs, shrubs, trees), and sometimes zero-grazing pens (Fig. 3). Biological material is also collected on moderately sloping land from contour hedgerows along terraced ridges and intercropped hedgerows. Plants growing in these areas help to soil erosion, conserve soil nutrients, and limit competition with crops for water and sunlight [22]. Fuel wood is typically sourced from trees around homes (in 41% of cases), from community forests and national parks (71%), or bought from traders (19.4%). In 25% of cases, fuel wood comes from two or three sources, but in some cases, material cultivated around homes is sufficient.

### Processing and administration

Of the 75 species recorded in the study area, the most commonly used growth forms are trees (51% of species), herbs (24%), and shrubs (16%). Ten parts of the plant are used—most commonly the leaf (23%), fruit or stem (14%), flower (10%), or bark (9%). Other parts used are the root, flower, bark, seed, latex, shoot, and resin. The entire plant is rarely used (3%). Generally, fresh plant parts are collected and used immediately. Alternatively, plants are stored in the shade or dry places in their original form, powdered, or used as an ash. Plants are consumed directly, roasted, juiced or pickled, or applied externally using the paste of leaves or milky latex. Administration of most medicinal plants is via decoction (mashing, and boiling the plant in water to extract oils, volatile organic compounds, and other chemical substances), although dermatological ailments are usually treated topically. For example, the leaves of *Azadirachta indica* (Margosa tree) are used to wash the skin to treat scabies [54–56].

Administration of biological material varies both daily and seasonally. For example, on Tuesdays and Thursdays, women practice a ritual that involves grinding and eating the root of *Mimosa pudica* (Touch-me-not plant) or chew the stem of *Calotropis gigantea* (Crown flower) to promote their husbands’ wellbeing. Other species are regularly ingested, such as *Aegle marmelos* (Bengal quince), the leaves (“*beal patra*”) and fruit pulp of which are used as an offering to Lord Shiva [55, 56]; *Asparagus racemosus* (Asparagus), which is used to prepare alcohol in August; and *Paris polyphylla* (Herb paris), the fruit of which is used for worship in mid-April. Although most Community Forestry User Groups officially restrict the harvest of fuel wood between December–February and during festivals in November (e.g., *Daishan*, *Tihar*), in 48% of cases fuel wood is collected throughout the year.

### Knowledge of plant use

The Nepalese are a culturally diverse population, as such we set out to assess whether knowledge of use and maintenance of species on farms differed according to caste^2^. The caste system is a traditional classification system of 36 hereditary groups of hierarchical social classes, which are defined through a combination of elements of birthright, ethnicity, and financial acumen. This may determine one’s education, income, occupation, and social standing [57]. While recognizing social classifications are socially constructed and evolving, broadly defined there are four social classes if one follows the *Chaturvarnashram* model: Brahmin, Kshatriya, Vaishya, and Sudra.

**Fig. 4:**
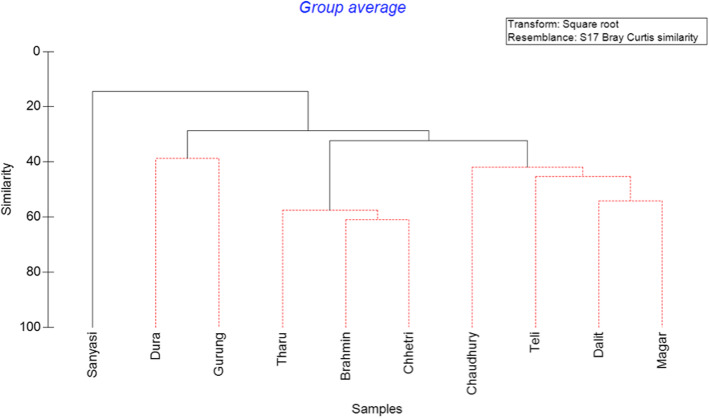
Cluster analysis of caste and plant species abundance. The figure shows knowledge is clustered around four statistically dissimilar groups. Black lines indicate relationships that are significantly supported, while red lines indicate no significant difference was detected

Of the ten castes are represented, SIMPROF tests identified four statistically different caste groups in similarity of knowledge: Chettri, Brahmin, and Tharu had the most diverse (*H*′) knowledge of plant uses and had a 40% similarity in knowledge of species. Dura and Gurung had a 30% similarity in knowledge, as did Chaudhury, Teli, Dalit, and Magar, while Sanyasi had a unique knowledge base endogenous to the region (Fig. 4). No significant relationships between absolute (*F*_(9.30)_ = 0.87, *p* = 0.56) or proportional abundance (*x*^2^_(167)_ = 155.65, *p* = 0.108) of plant species and caste were found. All three analyses indicated Brahmin, Tharu, and Chhetri generally had the most diverse knowledge, but Sanyasi, Magar, and Chaudhury had higher scores when controlling for the number of respondents representing each caste in the sample (Fig. 4). Males reported more plants (10 ± 0.87) than women (9.09 ± 1.51); however, the difference in the knowledge of plants between genders was not significant (*x*^2^(15) = 15.45, *p* = 0.420). No significant differences in species diversity according to age (*x*^2^(330) = 356.78, *p* = 0.149) were observed (Table 2).

**Table 2:** The number of useful plants reported by female and male informants in the Terai Plains of Nepal (Values show the mean ± SE).

No. medicinal plants reported	Female (age category in years)			Male (age category in years)		
	18–39 years	≥ 40 years	Total	18–39 years	≥ 40 years	Total
	(33.57 ± 1.54)	(50.73 ± 1.64)	(38.73 ± 1.47)	(35.86 ± 0.88)	(43.75 ± 1.93)	(46.59 ± 2.01)
1–5	1	0	1	2	1	3
6–10	4	2	6	2	14	16
11–15	1	2	3	3	4	7
16–20	1	0	1	0	2	2
21–25	0	0	0	0	0	0
26–30	0	0	0	0	1	1
Total	7	4	11	7	22	29

### Reasons for use

Interviews revealed that use depends on availability of nearby resources and alternatives or supplements (e.g., synthetic building material, electricity infrastructure), affordability, available travel or collection time, effectiveness of use (e.g., for medicinal purposes), and appropriateness based on traditional customs, spiritual beliefs, and livelihood strategies. Plants are generally used for domestic purposes in the household economy, rather than for commercial sale. Key plant species and their major uses are presented in Table 3.

**Table 3:** Key plant species and their major uses in Central and Western Terai Plains of Nepal

Scientific name, authority name, voucher number	Family	English name	Common name	Type	Part used	Uses	Reference, status
*Achyranthes bidentate* Blume N110-36	Amaranthaceae	Hill chaff flower	*Datiwan* (N)	Shrub	Leaves, stem	• Household (toothbrush–stem)	IUCN (2004) [55], Singh et al. (2012) [56], Accepted name
						• Spiritual (In *Teej* festival, women use leaves to welcome the monsoon season, bathe in the leaves as an act of purification, in Chitwan harvested for festival “*Rishi Tarpani*”)	
*Acorus calamus* L. N106-37	Acoraceae	Sweet flag	*Bojho* (N)	Herb/Monocot	Root	• Medicine (cough, common cold, increases internal heat, chronic fever, juice of root given orally or chewed to clear the throat)	IUCN (2004) [55]: 47, Accepted name
*Aegle marmelos* L. Corrêa N304-01	Rutaceae	Bengal Quince	*Bael, Bel* (N)	Tree	Root, leaves, fruit	• Medicine (Three leaves are used weekly for diabetes, root juice given orally for asthma and cold)	IUCN (2004) [55]: 32, Singh et al. (2012) [56], Accepted name
						• Spiritual (fruit pulp offered to Lord Shiva, women e fast on Mondays and use it the leaf with the Dubo leaf for the wellbeing of husbands, Newari’s marry the fruit)	
*Albizia lebbeck* L. Benth. N304-03	Leguminosae	Black siris	*Kalo Siris* (N)	Tree	Bark, stem, leaves, seeds	• Timber	Mishra (2013) [58], Accepted name
						• Compost	
						• Fodder (sweet seeds)	
						• Medicine (reduces inflammation using bark)	
						• Fuel (cooking)	
*Aloe vera* L. Burm. f. N103-05	Xanthorrhoeaceae	Aloe vera	*Kumari, Ghyekumari* (N)	Herb	Leaves, root	• Medicine (Cooling burns on skin, jaundice)	Govt of Nepal (2014) [59], Accepted name
						• Cosmetic (face cream)	
*Artemisia vulgaris var. indica* (Willd.) Hassk. N209-06	Compositae	Mugwort	*Tite pati* (N), *Pati* (Th)	Herb	Leaves, flowers, roots	• Spiritual (flower used for worship offering)	IUCN (2004) [55]: 187, Singh et al. (2012) [56], Accepted name
						• Medicine (stomach pains, digestion—tender root, antibiotic for cuts)	
						• Fodder (leaves)	
						• Compost manure	
						• Pesticide	
						• Rice diseases	
*Artocarpus heterophyllus* Lam. N101-07	Moraceae	Jackfruit	*Rukh Katahar* (N), *Badahar* (Th)	Tree	Fruit, stem, leaves	• Food (fruit, seed is eaten, roasted or cooked as a vegetable)	Storrs and Storrs (1998) [54]: 40, Accepted name
						• Pesticide	
						• Household (wood is used for a pot used to make yoghurt, called “*taki*”)	
*Asparagus racemosus* Willd. N102-08	Asparagaceae	Asparagus	*Kurilo* (N), *Satavari, Santawar* (Th)	Herb	Root	• Medicine (nutrition of humans/animals, paralysis, root powder given orally to increase lactation and tonic after delivery)	Singh et al. (2012) [56], Dangol (2005), Accepted name
						• Food (alcohol)	
*Azadirachta indica* A. Juss. N106-10	Meliaceae	Margosa tree	*Neem* (N), *Topre voltabre* (Th)	Tree	Leaves, bark	• Medicine (cough, gastritis, arthritis, wounds—and tender twigs paste, high blood pressure and high uric acid—fresh leaves given orally, scabies—fresh leaves used to wash ski, fever—bath in water boiled with neem leaves, pneumonia—boil 2-3 leaves and drink water)	IUCN (2004) [55]: 129, Storrs and Storrs (1998) [54]: 44, Singh et al. (2012) [56], Accepted name
						• Pesticide	
						• Household (young stem used to brush teeth)	
*Bombax ceiba* L. N204-12	Malvaceae	Silk cotton tree	*Simal* (N), *Semar* (Th)	Tree	Flowers, root	• Household (mattresses, cotton)	IUCN (2004) [55]: 168, Singh et al. (2012) [56], Accepted name
						• Timber	
						• Medicine (root decoction is given as tonic, anti-dysenteric, urinary infections)	
*Borassus flabellifer* L. N303-13	Arecaceae	Coconut	*Nariwal* (N)	Tree	Bark, fruit	• Food (fruit)	WCFP (2015), Accepted name
						• Spiritual (Coconut with milk inside is a holy offering)	
						• Timber	
*Bunium persicum* (Boiss.) B. Fedtsch N204-20	Apiaceae	Black cumin	*Himali, Kalo jeera* (N)	Herb	Seeds	• Medicine (cold—infused)	USDA (2015), Accepted name
						• Fuel (cooking)	
*Butea monosperma* (Lam.) Taub. N108-15	Leguminosae	Flame of the forest	*Palans, Dhak, Paras* (N)	Tree	Entire tree	• Medicine (diuretic)	IUCN (2004) [55]: 140, Accepted name
						• Pesticide to kill mosquitos	
						• Spiritual (Tree represents the God of Fire and flowers are used to worship Shivatri in Hinduism tree was used to achieve enlightenment in Theravada Buddhism)	
						• Household (leaves pieced together to make a leaf-plate (*patravali*))	
*Calotropis gigantean* (L.) Dryand N108-11	Apocynaceae	Crown flower	*Aak, Aankh* (N), *Madar* (Th)	Shrub	Latex	• Medicine (Massage milk into sprained areas (muscles/joints)	Singh et al. (2012) [56], Accepted name
						• Disservice (if the milk goes into the eye it can damage)	
						• Spiritual (referred to as the Tuesday bush)	
*Cannabis sativa* L. N201-16	Cannabaceae	Cannibis	*Bhang* (N)	Shrub	Entire plant	• Food	Mishra (2003) [58], Accepted name
						• Medicine (headache, dysentery, asthma)	
						• Veterinary (diarrhea, abscess of goats)	
*Carica papaya* L. N204-17	Caricaceae	Papaya	*Mewa* (N), *Papita, Larmewa* (Th)	Tree	Latex, fruit	• Household (Milk latex used to make soap)	Kerkhoff (2003) [60], Singh et al. (2012) [56], Accepted name
						• Medicine (muscle pain, tiredness—fruit infused)	
						• Fuel (cooking)	
*Centella asiatica* (L.) Urb. N109-22	Apiaceae	Indian pennywort	*Ghod tapre* (N), *Ghortapya, Boltapre, Bhatbhat* (Th)	Herb	Entire plant	• Medicine (fever, chest and urinary tract infections, typhoid blood purifier, pneumonia—infused)	IUCN (2004) [55]: 76, Singh et al. (2012) [56], Accepted name
*Cinnamomum tamala* (Buch.-Ham.) T.Nees & Eberm. N202-23	Lauraceae	Indian cassia	*Tejpaat* (N), *Dalchini* (Th)	Tree	Leaves	• Spiritual (ritual importance during festivals)	IUCN (2004) [55]: 184, Accepted name
						• Medicine (sore throat—infused)	
						• Food (Leaves eaten, imparting a strong cassia- or cinnamon like aroma to dishes)	
*Citrus hystrix* DC. N303-24	Rutaceae	Makrut lime	*Nibuwa* (N)	Tree	Stem	• Timber	Kerkhoff (2003) [60], Accepted name
*Colocasia affinis* Schott N201-25	Araceae	Taro or Elephant ear	*Pindalu, Karkalo* (N), *Ghuiya* (Th)	Herb	Stem, leaves, root, latex	• Food (leaves, root, stem or corn eaten as a vegetable)	Singh et al. (2012) [56], Accepted name
						• Medicine (latex sooths itching)	
*Crateva unilocularis* (Buch.-Ham.) N101-28	Capparaceae	Garlic pear	*Siplikan* (N)	Tree	Fruit	• Medicine (rheumatism, kidney, bladder stones, tonic)	IUCN (2004) [55]: 171, Govt of Nepal (2014), Accepted name
						• Food (vegetable high in iron)	
*Curcuma angustifolia* Roxb. N101-29	Zingiberaceae	Black turmeric	*Haledo* (N)	Herb	Leaves, root	• Medicine (stomach ailments, natural antibiotic for colds—infused)	IUCN (2004) [55]: 82, Accepted name
*Cuscuta reflexa* Roxb. N402-30	Convolvulaceae	Mistletoe	*Aakashbeli* (N) *Baora* (Th)	Climber	Entire plant	• Medicine (fever -juice of plant given orally, rheumatism and jaundice—plant paste applied externally)	Singh et al. (2012) [56], Accepted name
*Cymbopogon flexuosus* (Nees es Steud.) W. Watson N206-31	Poaceae	Lemongrass	*Ushir* (N)	Grass	Leaves	• Medicine (aromatic stimulant, oil, colds- infused)	Govt of Nepal (2014), Accepted name
*Cynodon dactylon* (L.) Pers. N109-32	Poaceae	Bermuda or Dog tooth’s grass	*Dubo* (N), *Dub* (Th)	Herb	Entire plant	• Spiritual (green grass used to decorate garlands)	Singh et al. (2012) [56], Accepted name
						• Fodder	
*Dalbergia sissoo* DC. N404-39	Leguminosae	Indian rosewood	*Sisau (N) Sisava* (Th)	Tree	Bark, stem	• Fodder	IUCN (2004) [55]: 173, Accepted name
						• Timber (house, furniture)	
						• Fuel (cooking)	
*Dendrocalamus strictus* (Roxb.) Nees N207-19	Poaceae	Feathery bamboo	Baas (N)	Grass	Entire plant	• Timber (Fences for farms and livestock, windbreaks, houses)	Storrs and Storrs (1998) [54]: 308, Accepted name
						• Fuel (cooking)	
*Duranta erecta* L. N108-40	Verbenaceae	Golden dewdrop or Forgetmenot	*Nil kanda* (N)	Shrub	Flowers	• Ornamental (grown for decoration along roadsides)	Storrs and Storrs (1998) [54]: 310, Accepted name
*Eucalyptus robusta* Sm. N202-41	Myrtacaeae	Gum tree	*Masala* (N)	Tree	Stem	• Timber (furniture)	Storrs and Storrs (1998) [54]: 117, Accepted name
*Chromolaena odorata* (L.) R. M. King & H. Rob. N210-26	Compositae	Croftonweed or sticky snakeroot	*Banmara* (N) *Banmasa, Banmari* (Th)	Shrub	Flowers	• Invasive weed, plant around the house and it prevents snakes from coming in, flower is used for worship, invasive species seeds used for making antibiotic, fodder, pesticide, compost	USDA (2015), Accepted name
*Euphorbia hirta* (L. N406-42	Euphorbiaceae	Asthma plant	*Dudhe jaar* (N), *Doodhe jaare* (Th)	Herb	Entire plant	• Fodder	Singh et al. (2012) [56], Accepted name
						• Cosmetic (seed used to wash hair)	
						• Medicine (plant juice applied to wounds)	
*Euphorbia royleana* Boiss. N101-43	Euphorbiaceae	Cactus, Royles’ or Sullu spurge	*Siundi* (N)	Tree	Entire plant	• Timber (fences, have thorns to protect from wild animal raids)	IUCN (2004) [55]: 175, Accepted name
						• Medicine (ground material is used for coughs)	
*Ficus benghalensis* L. N201-44	Moraceae	Banaya fig, or Indian Banyan	*Bar* (N), *Bargad* (Th)	Tree	Bark, latex	• Medicine (milky latex applied on muscular pain, infused barks given orally for diabetes)	IUCN (2004) [55]: 28, Singh et al. (2012) [56], Accepted name
*Ficus racemosa* L. N301-45	Moraceae	Cluster fig, or Indian fig tree	*Badar, Gular* (N), *Dumri* (Th)	Tree	Fruit, stem	• Food (fruit)	Storrs and Storrs (1998) [54]: 129, Mishra (2013), Accepted name
						• Timber	
*Ficus religiosa* L. N107-46	Moraceae	Bhodi or Peepal tree	*Pipal* (N)	Tree	Entire plant	• Medicine (cuts, cough—bark)	IUCN (2004) [55]: 145, Storrs and Storrs (1998) [54]: 129, Accepted name
						• Spiritual (sacred tree in Hinduism, Jainism and Buddhism)	
*Ficus auriculata* Lour. N301-21	Moraceae	Roxburgh fig	*Ninmaro* (N)	Tree	Leaves, fruit	• Fodder	IUCN (2004) [55], Accepted name
						• Food (fruit)	
*Garuga pinnata* Roxb. N104-14	Bursaraceae	Garuga	*Ramsing, Dabdabi* (N), *Dabdaai* (Th)	Tree	Leaves	• Fodder	Mishra (2003) [58], Accepted name
						• Veterinary (medicine)	
*Hibiscus rosa-sinensis* L. N101-47	Malvaceae	Chinese hibiscus	*Rakta puspi, Japa puspi* (N)	Shrub	Flowers, seeds, roots	• Spiritual (flowers placed in entrance to homes as offerings)	Ross (2003), Accepted name
						• Food (infusion)	
*Jacaranda mimosifolia* D. Don N101-48	Bignoniaceae	Jacaranda	*Jacaranda* (N)	Tree	Flowers	• Ornamental • Spiritual (flowers placed in entrance to homes as offerings)	IUCN (2004) [55]: 156, Accepted name
*Jatropha curcas* L. N208-49	Euphorbiaceae	Physic nut	*Sajiwan, Sajiba* (N) (Th)	Tree	Seeds, stems	• Timber (fences, livestock pens)	Storrs and Storrs (1998) [54]: 314, Accepted name
						• Household (biodiesel produced on a small-scale, seeds used for burning candles, brushing the teeth—shoot)	
						• Medicine (prevents gingivitis)	
*Justicia adhatoda* L. N408-50	Acanthaceae	Malabar nut	*Asuro* (N) *Osuro, Ross* (Th)	Shrub	Entire plant	• Medicine (headaches, dizziness, coughs, malaria treatment- dried powder of entire plant, swelling—grind leaves and drink)	IUCN (2004) [55]: 21, Storrs and Storrs (1998) [54]: 161, Accepted name
						• Pesticide	
						• Compost for seedlings in rice fields	
						• Timber (windbreaks, fences, houses)	
						• Fodder	
						• Food (vegetable)	
*Lannea coromandelica* (Houtt.) Merr. N206-51	Anacardiaceae	Indian ash tree	*Jinghat* (N)	Tree	Leaves	• Fodder	Mishra (2003) [58], Flowers of India (2015), Accepted name
*Lantana camara* L. N401-52	Verbenaceae	Lantana	*Ban fanda, Masino kanda* (N)	Herb	Flowers	• Spiritual (ornamental flower)	Ghosh (2012) Ethnobotanical Soc of Nepal (2015), Accepted name
						• Disservice (invasive species, toxic to livestock)	
*Leucaena leucocephala* (Lam.) de Wit N103-33	Leguminosae	Leucaena	*Ipil-ipil* (N) (Th)	Tree	Stem, leaves	• Fodder (for goats)	Kerkhoff (2003) [60], Storrs and Storrs (1998) [54]: 4, Accepted name
						• Fuel (cooking)	
						• Soil (erosion prevention along riverbanks)	
*Litchi chinensis* Sonn. N406-53	Sapindaceae	Litchee	*Litchi* (N) (Th)	Tree	Fruit	• Food (fruit)	Kerkhoff (2003) [60], Accepted name
						• Medicine (tonic, bites of animals)	
*Mangifera indica* L. N104-54	Anacardiaceae	Mango	*Amp* (N)	Tree	Fruit, bark	• Food (fruit)	Storrs and Storrs (1998) [54]: 179, Accepted name
						• Shade	
						• Medicine (stomach pains, fever—bath in cool water)	
						• Fuel (heating)	
						• Timber (house, furniture)	
*Melanochyla caesia* (Blume) Ding Hou (syn. *Semecarpus anacardium* Blume) N408-68	Anacardiaceae	Marking nut tree	*Bhalayo* (N)	Tree	Fruit	• Medicine (fruit and nuts used for skin allergies, ash used for scorpion or snake bites)	Storrs and Storrs (1998) [54]: 261, Unresolved name
*Melia azedarach* L. N110-55	Meliaceae	Persian lilac	*Bakaino/a* (N), *Bakain* (Th)	Tree	Leaves, Flowers and fruit	• Fodder	Kerkhoff (2003) [60], Storrs and Storrs (1998) [54]: 179, Accepted name
						• Pesticides (leaves stored in airtight container and left to decay, and sprayed on leaves)	
						• Fuel (cooking)	
						• Medicine (blood purifier–root, nausea, worms, leaf paste applied for scabies)	
*Mentha arvensis* L. N304-27	Labiatae	Peppermint	*Pudina* (N), *Baabari* (Th)	Herb	Entire plant	• Medicine (nausea, painkiller for headaches, neck pain, joint/back pain—applied externally with four parts oil and one part of crushed mint)	Singh et al. (2012) [56], Accepted name
						• Food (pickle)	
*Michelia champaca* (L.) Baill. Ex Pierre N302-35	Magnoliaceae	Champak	*Champaa* (N), *Chuwa* (Th)	Tree	Flowers, leaves	• Medicine (blood pressure, diabetes)	Storrs and Storrs (1998) [54]: 184, Accepted name
						• Spiritual (offering in temples and homes)	
*Mimosa pudica* L. N301-56	Leguminosae	Touch-me-not or Humble plant	*Lajjawati* (N)	Shrub	Leaves	• Medicine (constipation, extended stomach—roots ground and is mixed with Tulsi, Rudilo, Beloti leaves, Titepati and the root of Datiwan—eaten on Tuesdays and Thursdays)	IUCN (2004) [55]: 118, Govt of Nepal (2014), Accepted name
						• Fodder	
*Musa paradisiaca* L. N203-57	Musaceae	Banana	*Kera* (N) (Th)	Shrub	Entire plant	• Food (fruit)	Kerkhoff (2003) [60], Singh et al. (2012) [56], Accepted name
						• Soil (soil potassium input)	
						• Household (leaf used to place meat which is cut on top)	
						•Medicine (dysentery – fruit roasted)	
*Neolamarckia cadamba* (Roxb.) Bosser N401-58	Rubiaceae	Kadamba tree	*Kadam, Kadamba* (N)	Tree	Stem, leaves	•Timber (low-grade wood used for light construction)	WCFP (2015), Accepted name
						• Fuel (cooking, heating)	
						• Medicine (mouth gargle—extract of leaves)	
						• Fodder (cattle)	
						• Spiritual (in Hinduism the sacred couple of Shiva and Parvati came to *Sahyadr,* and gave birth to a child under the Kadam tree)	
*Nyctanthes arbor-trisis* L. N304-59	Oleaceae	Coral jasmine	*Parijat, Paarijaat* (N), *Raatki rani, Hasna* (Th)	Shrub	Flowers, seeds, leaves	• Spiritual (flower used in several Hindu religious stories and is often related to the Kalpavriksha)	Govt of Nepal (2012), Accepted name
						• Medicine (cough, asthma, diuretic)	
*Ocimum tenuiflorum* L. N103-60	Lamiaceae	Holy or Sacred basil	*Krishna tulsi* (N), *Kalo tulasi* (Th)	Herb	Entire plant	• Medicine (cough, fever, immune booster—infused daily with turmeric)	IUCN (2004) [55]: 189, Singh et al. (2012) [56], Accepted name
						• Spiritual (grown in homes for daily offering)	
						•Soil (ground cover to retain moisture)	
*Oxalis corniculata* L. N201-76	Oxalidaceae	Creeping sorrel	*Carii Amilo, Caremalaa* (N)	Creeper	Flowers, leaves	• Food (leaves edible, with tangy taste of lemon)	IUCN (2004) [55]: 49, Accepted name
						• Disservice (invasive weed in fallow land)	
*Bryophyllum pinnatum* (Lam.) Oken N210-18	Crassulaceae	Life plant, Air plant, Miracle leaf	*Ajambari/a* (N)	Herb	Root, leaves, stem	• Compost manure (leaves)	Mandal et al. (2013) [61], Accepted name
						• Timber (fences, canals)	
						• Disservice (if the leaves are eaten by the livestock they die)	
						• Fuel (cooking)	
*Paris polyphylla* Sm. N301-61	Melanthiaceae	Herb paris	*Langokloti, Satuwa* (Th)	Herb	Leaves, fruit	• Medicine (fever, headache—mix with tobacco)	Madhu et al. (2010), Accepted name
						• Veterinary (medicine for livestock)	
						• Spiritual (Gurungs (a caste) harvest the fruit on Tuesdays of mid-April)	
*Phyllanthus emblica* L. N102-62	Phyllanthaceae	Indian gooseberry	*Amala* (N), *Aura* (Th)	Tree	Leaves, bark, fruit	• Food (fruit)	IUCN (2004) [55]: 16, Storrs and Storrs (1998) [54]: 209, Singh et al. (2012) [56], Accepted name
						• Medicine (gastric problems including dysentery, constipation, stomach tumors and back pain—bark juice given orally, sore throat and colds—decoction)	
						• Household (used in soaps and cosmetics)	
*Pogostemon benghalensis* (Burm. f.) Kuntze N304-34	Lamiaceae	Bengal pogostemon	*Rudhilo* (N)	Herb	Flowers, roots, leaves, shoots	• Medicine (temperature balance, sinus, cold, sedative, stimulant, styptic))	Dongol (2005), Accepted name
						• Compost manure	
						• Spiritual (flowers used for garlands)	
						• Household (patchouli / dilem essential oil)	
*Psidium guajava* L. N208-04	Myrtaceae	Guava	*Aamba, Belauti* (N) *Belaun* (Th)	Tree	Fruit, stem	• Food (fruit)	Kerkhoff (2003) [60], Accepted name
						• Fuel (cooking, heating)	
						• Medicine (throat, diarrhea, vomiting)	
*Pueraria tuberosa* (Willd.) DC. N105-63	Leguminosae	Indian kudzu	*Kudzu* (N) *Biralikand* (Th)	Climber	Root, fruit	• Medicine (relieves constipation, eases bowel movement, boosts immunity)	IUCN (2004) [55]: 46, Accepted name
*Rauvolfia serpentina* L. Benth. Ex. Kurz N107-64	Apocynaceae	Indian snakeroot	*Sarpagangha* (N)	Shrub	Flowers, roots	• Medicine (stomach pain, bowel disorder, dysentery, hypotension, sedative)	Govt of Nepal (2014), Accepted name
						• Spiritual (used in meditation)	
*Ricinus communis* L. N101-65	Euphorbiaceae	Castor oil	*Ander* (N) *Yamyam* (Th)	Shrub	Root, leaves, seeds	• Medicine (sprains – seed oil or leaves heated and massaged, rheumatic pain—given orally)	Storrs and Storrs (1998) [54]: 336, Singh et al. (2012) [56], Accepted name
*Salix* x *fragilis* L. N207-66	Salicaceae	Willow	*Bainsh* (N)	Tree	Entire plant	• Ornamental	Aziz (2007), Accepted name
						• Timber	
						• Shade	
						• Fodder	
						• Medicine (fever)	
*Sapindus mukorossi* Gärtn. N301-67	Sapindaceae	Soap nut	*Rittha* (N)	Tree	Fruit, leaves	• Household (nut used for soap, leaves used for baskets)	IUCN (2004) [55]: 155, Accepted name
						• Food	
						• Medicine (epilepsy, salivation)	
*Shorea robusta* Gärtn. N403-74	Dipterocarpaceae	Sal tree	*Sal* (N) *Sakhuwa* (Th)	Tree	Root, bark, resin, seed	• Timber (hardwood for furniture, buildings)	Storrs and Storrs (1998) [54]: 264, IUCN (2004) [55]: 158, Kerkhoff (2003) [60], Accepted name
						• Fuel (cooking)	
						• Medicine (Diarrhea, bloody dysentery—administered orally)	
*Syzygium cumini* (L.) Skeels N201-75	Myraceae	Black plum or Indian blackberry	*Phader* (N) *Jamun, Jamuna* (Th)	Tree	Fruit, bark, seed, leaf	• Medicine (Diarrhea, indigestion, headaches, constipation—fruit given orally, bronchitis—bark, leaf and seed, powder is given orally to reduce sugar levels in blood for diabetes and to improve the heart)	IUCN (2004) [55]: 90, Ghosh (2012), Accepted name
						• Fodder	
						• Food (fruit eaten or fermented for alcohol)	
*Terminalia bellirica* (Gärt.) Roxb. N102-69	Combretaceae	Barro or Bekkeric myrobalan	*Barro* (N) *Baheda* (Th)	Tree	Seeds	• Household (soap, hair oil)	IUCN (2004) [55]: 29, Accepted name
						• Medicine (gastric, dry cough, cold—roast the seed and chew)	
*Terminalia chebula* Retz. N405-70	Combretaceae	Yellow or Chebulic myrobalan	*Rohini* (N)	Tree	Fruit	• Food (fruit, young leaves pickled or made into preserves when boiled and added with sugar)	Bhattarai et al. (2006) [14], Accepted name
						• Fuel	
						• Fodder	
						• Medicine (compounds of chebulic acid inhibit the growth of malignant tumors, cures blindness)	
*Themeda triandra* Forssk. N201-09	Poaceae	Kangaroo or Rui grass	*Khari* (N), *Kiyar* (Th)	Tree	Stem, leaves	• Timber (furniture)	Storrs and Storrs (1998) [54]: 78, Accepted name
						• Medicine (pain in the body)	
						• Household (clothing dye)	
						• Fodder	
						• Fuel (cooking)	
*Thysanolaena latifolia* (Roxb. ex Hornem.) Honda N304-02	Poaceae	Broom or Bouquet grass	*Amriso* (N)	Herb	Leaf, stem	• Household (used to make brooms)	Shankar et al. (2001), Accepted name
*Tinospora sinensis* (Lour.) Merr. N204-71	Menispermaceae	Guduchi	*Gurjoo* (N)	Shrub	Leaves	• Medicine (fever, maintains thermal balance)	IUCN (2004) [55]: 80, Govt of Nepal (2014), Accepted name
						• Fodder	
*Vitex negundo* L. N107-72	Lamiaceae	Chaste tree	*Simali* (N)	Shrub	Leaves	• Medicine (stomach pain, juice given orally)	IUCN (2004) [55]: 169, Singh et al. (2012) [56], Accepted name
*Zanthoxylum armatum* DC. N205-38	Rutaceae	Winged prickly ash	*Timur* (N)	Tree	Fruit	• Medicine (toothache, common cold, cough, fever—particularly for children)	Govt of Nepal (2014), Accepted name
*Zingiber officinale* Roscoe N207-73	Zingiberaceae	Ginger	*Ardhrakam* (N)	Herb	Root	• Medicine (sore throat, cough)	USDA (2013), Accepted name

The table summarizes the scientific name, family, common name, type, part used, and uses of the 76 species identified. Plant specimens were collected using standardized sampling procedures and farmers were asked to free-list vernacular names and other information (*n* = 40 sites). Scientific and English names and family were identified or corroborated using reference material (listed in column 8). All of the data represents original findings. The status of plants is determined according to the World Flora Online Consortium [62].

#### Multipurpose woodlots

Multipurpose woodlots (known as “*Bagaincha*” (N), or “*Fulbari*” (Th)) provide fuel, fodder, timber, food, fertilizers, pesticides, and control erosion. In total, 33 multipurpose tree species found on farms and around homesteads were recorded through visual observation. The average number of trees grown around farms is 6.03 ± 4.35, ranging from 1 to 26. Ten tree species are used for fodder, six of which are found around homesteads (i.e., *Dalbergia sisoo* (Indian rosewood) (62.5%), *Melia azedarach* (Persian lilac) (50%), *Leucaena eucocephala* (Leucaena) (42.5%), *Garuga pinnata* (Garuga) (12.5%), *Artocarpus interga/heterophyllus* (Jackfruit), *Artemisia indica* (Mugwort)). Fifteen tree species are used for food (mostly fruit), ten of which are found around homesteads (e.g., *Mangifera indica* (Mango), *Syzygium cumini* (Black plum), *Psidium guajava* (Guava) and *Phyllanthus emblica* (Indian gooseberry)). Three tree species are used to improve soil nutrient levels and prevent erosion, two of which are found around homesteads (i.e., *Leucaena leucocephala* (Leucaena), *Albizia lebbeck* (Black siris)). Four tree species are used for pesticides, all of which are found around homesteads (i.e., *Melia composite*/*azedarach* (Persian lilac), *Azadirachta indica* (Margosa tree), *Artocarpus heterophyllus* (Jackfruit), *Artemisia indica* (Mugwort)). Fourteen tree species are used for fuel wood, nine of which are found around homesteads. Fifteen tree species are used for timber, 11 of which are found around homesteads [54–56].

#### Energy use

The top ten species used for fuel wood are *Shorea robusta* (used by 65% of households), *Dalbergia sisoo* (Indian rosewood) (62.5%), *Melia azedarach* (Persian lilac) (50%), *Dendrocalamus strictus* (Bamboo), *Leucaena leucocephala* (Leucaena) (42.5%), *Mangifera indica* (Mango), *Jatropha curcas* (Physic nut), *Psidium guajava* (Guava), *Phyllanthus emblica* (Indian gooseberry), and *Garuga pinnata* (Garuga) [58]*.* Some species of wood have cultural significance, used especially during festivals or for cremation (refer to “Cultural use” section). Wood is the main source of household energy (97%), with electrical connections and frequent power outages. However, wood is not the only source of energy for these households: biogas (i.e., ox, cow, or human) (70%), liquefied petroleum gas (22%), solar (8%), crop residue (e.g., maize husks (8%)), or kerosene, candles, or battery lamps for lighting are also used.

#### Medicinal use

The collection of medicinal and aromatic plants is vital to Tharu human and animal healthcare, in combination with modern remedies. Plant medicine is preventative, curative, soporific, or stimulatory. Respondents reported 61 ailments that are treated with medicinal plants, including gastro-intestinal, dermatological, cardio-vascular, ureno-genital, respiratory, skeleto-muscular, mental disorders, or dental, eye, ear, nose, throat, birthing, or lactation issues. Medicinal plants are most commonly used for fever (*n* = 9), cough (*n* = 9), and cold (*n* = 8) and are often mixed in combination. For example, for constipation and a distended stomach, the root of *Mimosa pudica* (Touch-me-not plant) [55, 59] is ground and mixed with the root of *Achyranthes bidentata* (Hill chaff flower) [55, 56], and the leaves of *Pogostemon benghalensis* (Bengal pogostemon) [63], *Psidium guajava* (Guava) [60], and *Artemisia indica* (Mugwort) [55, 56]. Many remedies are influenced by *Ayurveda* used in India, and the *Bhaidya* system used in far-western Nepal [35]. Table 4 presents the plants used to remedy 61 ailments, with number of uses and taxa.

**Table 4:** Ailment categories of medicinal plants identified during interviews

Ailment category	Biomedical term	Species used	No uses	No taxa
Gastro-intestinal illness	Stomach pain	*Magnifera indica*, *Artemisia indica*, *Vitex negundo*, *Acorus calamus*, *Curcuma angustifolia*	11	5
	Dysentery	*Musa paradisiaca*, *Cannabis sativa*, *Shorea robusta*, *Phyllanthus emblica*, *Acorus calamus*		5
	Intestinal worms	*Melia azedarach*		1
	Diarrhea	*Psidium guajava*, *Shorea robusta*, *Syzygium cumini*		3
	Indigestion	*Artemisia indica*, *Syzygium cumini*		2
	Vomiting	*Psidium guajava*		1
	Nausea	*Melia azedarach*, *Mentha arvensis*		2
	Gastritis	*Azadirachta indica*		1
	Constipation	*Syzygium cumini*, *Phyllanthus emblica*, *Acorus calamus*, *Pueraria tuberosa*, *Mimosa pudica*		5
	Stomach tumors	*Phyllanthus emblica*		1
	Extended stomach	*Mimosa pudica*		1
Fever	Fever	*Magnifera indica*, *Ocimum tenuiflorum*, *Cinnamomum tamala*, *Centella asiatica*, *Acorus calamus*, *Paris polyphylla*, *Cuscuta reflexa*, *Pogostemon benghalensis*, *Zanthoxylum armatum.*	3	9
	Typhoid	*Centella asiatica*		1
	Malaria	*Crateva unilocularis*, *Butea monosperma*		2
Dermatological disorders	Scabies	*Melia azedarach*, *Azadirachta indica*	8	2
	Cut	*Ficus religiosa*		1
	Skin allergies	*Melanochyla caesia*		1
	Scorpion/snake bites	*Melanochyla caesia*		1
	Burns	*Aloe vera*		1
	Styptic	*Pogostemon benghalensis*		1
	Wounds	*Euphorbia hirta*		1
	Itching	*Colocasia affinis*		1
Cardio-vascular/blood	Blood purifier	*Melia azedarach*, *Centella asiatica*	3	2
	Blood pressure	*Azadirachta indica*, *Michelia champaca*		2
	Jaundice	*Aloe vera*, *Cuscuta reflexa*		2
Ear*,* nose and throat	Throat	*Psidium guajava*, *Acorus calamus*, *Zingiber officinale*	4	3
	Salivation	*Sapindus mukorossi*		1
	Bronchitis	*Syzygium cumini*		1
	Sinus infection	*Pogostemon benghalensis*		1
Ureno-genital problems	Urinary tract infections	*Bombas ceiba*, *Centella asiatica*	5	2
	Diuretic	*Bombas ceiba*, *Butea monosperma*, *Nyctanthes arbor-trisis*		3
	High uric acid	*Azadirachta indica*		1
	Bladder stones	*Crateva unilocularis*		1
	Kidney stones	*Crateva unilocularis*		1
Respiratory diseases	Cough	*Terminalia bellirica*, *Azadirachta indica*, *Ficus religiosa*, *Ocimum tenuiflorum*, *Crateva unilocularis*, *Acorus calamus*, *Zanthoxylum armatum DC*, *Nyctanthes arbor-trisis*, *Zingiber officinale*	4	9
	Common cold	*Terminalia bellirica*, *Acorus calamus*, *Curcuma angustifolia*, *Ageratina adenophorum syn. Eupatorium adenophorum*, *Euphorbia royleana*, *Pogostemon benghalensis*, *Zanthoxylum armatum *, *Cymbopogon flexuosus*		8
	Asthma	*Cannabis sativa*, *Nyctanthes arbor-trisis*		2
	Pneumonia	*Azadirachta indica*, *Centella asiatica*		2
Skelto-muscular pain and swelling	Swelling	*Crateva unilocularis*	9	1
	Arthritis	*Azadirachta indica*		1
	Muscular pain	*Carica papaya*, *Ficus benghalensis*		2
	Neck pain	*Mentha arvensis*		1
	Headache	*Cannabis sativa*, *Syzygium cumini*, *Crateva unilocularis*, *Mentha arvensis*, *Paris polyphylla*		1
	Sprains	*Ricinus communis*, *Calotropis gigantea*		2
	Back pain	*Phyllanthus emblic*, *Mentha arvensis*, *Themeda Triandra*		3
	Inflammation	*Albizia lebbeck*		1
	Joint pain	*Mentha arvensis*, *Themeda Triandra Calotropis gigantea*		3
	Rheumatic pain	*Ricinus communis*, *Crateva unilocularis*, *Crateva unilocularis*, *Cuscuta reflexa*		4
Dental and eye disorders	Toothache	*Zanthoxylum armatum *	2	1
	Blindness	*Terminalia chebula*		1
Other	Epilepsy	*Sapindus mukorossi*	11	1
	Dizziness	*Crateva unilocularis*		1
	Stimulant	*Carica papaya*, *Pogostemon benghalensi*, *Cymbopogon flexuosus*		3
	Diabetes	*Ficus benghalensis*, *Syzygium cumini*, *Michelia champaca*		3
	Tonic	*Litchi chinensis*, *Crateva unilocularis*		2
	Immune booster	*Ocimum tenuiflorum*, *Pueraria tuberosa*, *Asparagus racemosus*		3
	Hypertension	*Acorus calamus*		1
	Sedative	*Acorus calamus*, *Pogostemon benghalensis*		2
	Tumors	*Terminalia chebula*		1
	Lactation	*Asparagus racemosus*		1
	Tonic after delivery	*Asparagus racemosus*		1

Respondents free-listed medicinal uses, which were then categorized into the listed medical terms. Many taxa are used for more than one ailment category. Categories used are based on a previous ethno-pharmacological study from the Terai [35], rather than that specified by informants.

#### Cultural use

Twenty-seven plants have cultural, spiritual, or religious significance and are associated with cultivation practices. Plants are used in symbolic rituals, offerings, religious occasions, marriage ceremonies, fasting, or acts of purification. For example, various flowers are used for worship in homes and temples as a ritualistic expression of reverence or adoration for deities, present daily offerings (e.g., *Jacaranda mimosifoliam* (Jacaranda) [55], *Lantana camara* (Lantana) [64], *Michelia champaca* (Champak) [54], and *Nyctanthes arbor-trisis* (Coral jasmine) [59]), or to make garlands for celebrations and welcome visitors (e.g., *Pogostemon benghalensis* (Bengal pogostemon) [63]). Women bathe in the leaves of *Achyranthes bidentata* (Hill chaff flower) during the festival of *Teej* to welcome the monsoon and as an act of purification [55, 56]. Traditionally, female Newaris (a caste) at the age of 6 months perform a symbolic marriage ceremony with *Aegle marmelos* (Bengal quince), and the size and morphology of the fruit is used to predict the character of the child’s future husband [55, 56]. Similarly, Hindu women fast on Mondays and offer the leaf of *Cynodon dactylon* (Dog tooth’s grass) [56] to Lord Ganesh for the wellbeing of one’s husband. On religious celebrations, the leaves of *Butea monosperma* (Flame of the forest) and *Shorea robusta* (Sal) [54, 55] are pieced together or used singly to make a leaf-plate on which to serve meals (“*patravali*” (N)). In many villages, the leaves of *Musa paradisiaca* (Banana) are used as a surface to cut communal meat shared with every village member for religious occasions [56]. Various trees considered sacred in Buddhist, Jain, and Hindu traditions are planted along roadsides, public areas, villages, and temples. For example, *Neolamarckia cadamba* (Kadamba) [65] is associated with a tree deity called “*Kadambariyamman*” and according to folklore, the sacred couple of Shiva and Parvati gave birth to a child under the tree. During a harvest festival on the eleventh moon day in the month of *Bhadra* (August/September), a twig is brought and worshipped in the courtyard of the house.

Three species of indigenous fig possess high religious value [66, 67]: *Ficus religiosa* (Pipal), *Ficus benghalensis* (Bar), and *Ficus racemosa* (Dumri) [54, 55]—and are often found in the center of villages next to each other or at shrines. In Buddhism, Buddha attained enlightenment underneath the Pipal tree [68]. *Ocimum tenuiflorum* (or *Ocimum sanctum*, *Tulsi* or Holy basil) [55, 56] is planted outside the homes of Hindus, often in masonry structures to indicate religious inclination of a family. The offering of its leaves is mandatory in the daily ritualistic worship of Lord Vishnu. The plant also has diverse healing properties and is used as an essential oil [69].

#### Veterinary use

For veterinary use, plant material is used for livestock medicine, fodder, shelter, and fences to deter wild animals. Livestock medicinal plants include *Cannibis sativa* (Cannibis) [58], used for diarrhea in ox, cattle, and buffalo; *Dendrocalamus strictus* (Bamboo) [54], used to treat abscesses in goats; and *Litchi chinensis* (Litchi) [60], used to treat animal bites. Common plants used for fences and pens include *Jatropha curcas* and *Dendrocalamus strictus* [54]. Plants are used to deter wild animals from raiding crops, such as *Euphorbia royleana* (Cactus spurge) [55] planted as a thorny fence to repel largest Asian antelope, the blue bull (*Boselaphus tragocamelus*), or *Eupatorium odoratum* (Crofton weed) [31] is planted around houses to deter snakes. Sixteen fodder species are collected on a daily basis.

#### Timber and building material use

*Shorea robusta* (Sal) is the most common species used for timber (used in 65% of cases), followed by *Dalbergia sisoo* (Indian rosewood) (62.5%), *Melia azedarach* (Persian lilac) (50%), and *Mangifera indica* (Mango) (32.5%). Timber products include both low-grade (e.g., *Neolamarckia cadamba* (Kadamba)) and hard woods (e.g., *Shorea robusta* (Sal)), used for constructing housing, livestock sheds, furniture, ladders, stairs, doors, and farm equipment. Eight types of grasses, herbs, and shrubs are similarly used for constructing houses. Around homes, plants are grown for windbreaks (e.g., *Dendrocalamus strictus* (Bamboo)), fences (e.g., *Euphorbia royleana* (Cactus spurge)), or shade (e.g., *Mangifera indica* (Mango)). Plants are used for ornamental purposes along roadsides (e.g., *Duranta erecta* (Golden dewdrop)), and homes (e.g., *Dendrocalamus strictus* (Bamboo)) [54].

#### Household use

Soap is made from the latex of *Carica papaya* (Paw paw), or nuts and seeds of *Sapindus mukorossi* (Soap nut), also used to weave baskets and mats. *Aloe vera* (Aloe vera) is used as a face cream and *Euphorbia hirta* (Asthma plant) is used to wash hair. *Bombax ceiba* (Silk cotton tree) is used to make cotton for mattresses and *Themeda Triandra* (Rui grass) is used as a clothing dye. Brooms are made of *Thysanolaena maxima* (Broom grass). Pots used for cultivating yoghurt, called “*taki*” (*N*), are made of *Artocarpus heterophyllus* (Jackfruit). The seed of *Jatropha curcas* (Physic nut) is burnt for fuel for transport and lighting, and the stem is used as a toothbrush. Plants are also used to make musical instruments, fishing baskets, toys, jewellery, and containers to carry wild harvested goods [54].

#### Wild edible plant use

Wild edible plants have noteworthy roles and contributions in Nepalese diets and food security, particularly for many indigenous, rural, ethnic, and marginalized people in Nepal [70, 71]. Wild edible plants are uncultivated plants found in the wild, with nutritious value for fulfilling dietary requirements. A number of agricultural crops, and their wild relatives and edible plants enrich the species and diversity of Nepal [70]. Twenty-five unique species are used as wild edible plants for food across the study. Vegetables are obtained from some plants, such as *Crateva unilocularis* (Garlic pear) which is high in iron, *Justicia adhatoda* (Malabar nut), and *Artocarpus heterophyllus* (Jackfruit). Others are used for pickle, such as *Mentha arvensis* (Peppermint), or *Terminalia chebula* (Yellow myrobalan). Plant’s edible seeds are either consumed after boiling or roasting, such as *Cannabis sativa* (Bhang) and *Artocarpus heterophyllus* (Jackfruit). Some plants are rich in nectar in the flower, such as *Hibiscus rosa-sinensis* (Chinese hibiscus). Some leaves are edible, such as *Oxalis corniculate* (Creeping sorrel). Others are used for fermenting substrates or alcohol, such as *Syzygium cumini* (Indian blackberry), or to make preserves when boiled with sugar, such as *Terminalia chebula* (Yellow myrobalan). Plants are also used for spices and herbs.

## Discussion

### Factors that incentivize the maintenance of ecosystem services in and around farms

Results offer evidence to support emerging claims that as agriculture intensifies and expands, farmers may increasingly play an important role in conservation beyond protected areas [32, 72]. Communities have a comprehensive understanding of the structure and function of the interconnected human-environmental systems in which they live, allowing them to secure necessities from ecosystem services [73, 74]. The utilization of local plant material is cost-effective, time-tested, situation-specific, practical, and flexible. Modifying or transforming existing norms and behaviors to deal with emerging stressors is common [75]. For example, traditional multi-cropping systems are maintained for various subsistence priorities, such as providing timber, fuel, medicine, organic fertilizer, and pesticides. Farmers maintain plants not only for economic reasons, but to conserve soil, prevent erosion, fix nitrogen, and decompose organic matter. They maintain trees, shrubs, and herbs to support photosynthesis, evapotranspiration, watershed regulation, carbon sequestration, and protect against crop raiding by animals [76]. Plants provide livestock fodder, and in turn, plowing power for cultivation, milk, meat, and manure [27]. Plants are cultivated in rotation plots, fallows, forests, home gardens, and are multistoried cropped to efficiently use space [74, 77]. Furthermore, plants have esthetic and ornamental value, interlinked with tradition, religious, and cultural heritage [78, 79].

In addition to these services, farmers manage “disservices.” For example, *Lantana camara* (Lantana) [80] is used as an ornamental and offering, but forms dense thickets, reduces farmland productivity, prevents the growth of new trees, and is toxic to livestock [81]. *Oxalis corniculata* (Creeping soral) is used for food, but is considered an invasive species that occupies fallow land [55]. *Calotropis gigantea* (Crown flower) has milky latex that is massaged into muscles to relieve sprains, but can cause blindness [56]. *Kalanchoe piñata* (Life plant) is used for compost and timber, but is also toxic for livestock [61]. As such, farmers’ practices are geared toward augmenting such services, and reducing disservices from and related to wild and tended plants in and around farms. This results in a restructuring of the agroecosystems in terms of diversity and abundance of wild plant species [82]. This is what can be termed “servicing ecosystems” [83]. Much of this agricultural knowledge and practice can be integrated into scientific knowledge.

### Ethnobotanical knowledge associated with the maintenance of ecosystem services on farms

Local knowledge, practice, and beliefs have accumulated over generations of living in particular environments, handed down through cultural transmission [84], including apprenticeships [35]. In this study, the average number of useful plants reported by informants of 18–39 years of age (8.64 ± 1.23) was lower compared to that reported by informants of ≥ 40 years (10.35 ± 0.93). This may suggest the beginnings of a potential loss of traditional ethnobotanical knowledge in the younger generation, which aligns with farmers’ perceptions of declining transmission of local knowledge. This is particularly significant because most botanical knowledge is acquired by young adults [85]. Young farmers have fewer elderly mentors, and agricultural livelihoods are increasingly discouraged in favor of new forms of service employment. Farmers associate this trend with observed declines or disappearance in the last 10 years of formerly tended plants, such as *Pueraria tuberosa* (Indian kudzu) [55], a climber, the root and fruit of which is used to relieve constipation and boost immunity; or *Kalanchoe piñata* (Life plant) [61], used for compost. This finding is consistent with previous findings showing [56] youngsters of both Tharu and migrant societies are less aware of plant use in the Terai, than previous generations. On the other hand, the higher number of plants reported by informants of ≥ 40 years of age corroborates the long-standing local belief that elders possess more botanical knowledge than other segments of society.

Ethnobotanical knowledge and practice within any culture varies according to geographic origin, residence, religion, age, gender, and ethnicity [86]. Nevertheless, some other explanations for differences in knowledge of plant use could be that (i) Brahmin and Chettri castes tend toward more secure land tenure and higher education, leading to a more diverse knowledge of plant use; (ii) the Tharu and Dura castes have a heritage of subsistence agriculture in the Terai, leading to a greater understanding of plant use; and (iii) the unique knowledge of the Sanyasi stems from Hindu Ayurvedic tradition [38]. However, the fact that social groups are geographically close, exposed to similar environments, and able to exchange knowledge readily could act as compounding factors [87].

### Medicinal plant use

Perhaps unsurprisingly, our analysis reveals that a large proportion of plants with documented uses are utilized as medicines: from dermatological to gastrological. Despite the reported increased number of healthcare clinics in the last 15 years, most respondents have a high reliance on, and prefer to use, plant-based remedies. Eighty percent of respondents consult herbalists or folk healers, often in combination with consultations with health workers (97%), visiting health clinics (80%), or hospitals (40%). This finding is supported by previous studies in other regions in Nepal, which suggest that two-thirds of the rural population relies on traditional herbal medicine [35, 56]. Reasons for this include cultural acceptance, a long history of traditional medicinal use, the affordability of traditional remedies compared to modern healthcare, and limited alternatives (i.e., the patient ratio in Nepal is 1:20,000 for medical professionals verses 1:100 for traditional healers [88–90]).

### Richness of plant use

When comparing the richness the plant use to other parts of Nepal, results were similar. For example, Kunwar et al. (2006) studied four districts in Nepal in Dolpa, Jumla, Humla, and Mustang. They found 84 common species, which compares to 76 common species that were found in four districts in the Terai [91]. Richness of plant use could not be compared with other countries due the fact that our study was not national, but focused on one agroecological zone of Nepal. When comparing the abundance of plants used, our study found fewer plants are used than reported in previous studies. For example, Dangol et al. (2017) found 396 wild edible plants that are collected [31]. Similarly, a study by Manandhar (1997) found 444 wild edible plants were harvested [92]. A study in southwestern China found 103 wild edible plants that are collected [93], while a study in southern Ethiopia found 66 species [94]. On the one hand, this may be due to a factor of the study design, which excluded cultivated edible plants in the livestock grazing and agricultural landscape. On the other hand, it may also be an indication of a decline of biodiversity in the face of global environmental change, as seen in many other parts of the world. It may also indicate that food resources are beoming increasingly dependent on markets in the Terai, especially in areas with convenient transportation and tourism activities. This finding resonates with other studies that suggest collecting wild edible plants is becoming increasingly rare, and the younger generation appears to be losing or even disgarding the traditional knowledge and use of wild edible plants. Nevertheless, eating wild plant foods can help relieve food shortages during drought years, reduce dependence on markets, or increase nutritional diversity [93].

### Building the evidence base

The continued contribution of plant agro-biodiversity to the maintenance of agroecosystem services will depend on how farmers gear management toward augmenting particular services, as well as the extent to which local knowledge is preserved, which will vary across types of crops, altitudes, seasons, and cultural needs [95]. In each context, evaluations of the optimal species mix or ecosystem type under multiple possible futures in particular locales are needed, along with approximations of substitutability. Future longitudinal research, across seasons or years, could consider how the knowledge and use of high-value species that provide ecosystem services is changing, such as species with high nutritional benefits, and commercialization viability (e.g., *Azadirachta indica* (Margosa tree), or *Artocarpus heterophyllus* (Jackfruit) [52, 53). Agricultural and agroforestry landscapes represent a largely unexplored source for pharmacological and phytochemical studies of new bioactive compounds for treating illnesses [35, 95–102]. Indeed, conserving medicinal plants for treating human illness is recognized as an important component of the Sustainable Development Goal (SDG) 3, and many opportunities exist to build evidence of the effectiveness of herbal remedies compared to modern methods [103, 104]. Relatedly, maintaining the integrity of the associated local ecological knowledge, considered critical in achieving many of the SDGs, is also essential. In this context, documentation may also provide a useful safeguard for the sovereignty of traditional knowledge and protect knowledge from being exploited and misappropriated by Big Pharma [96]. Moreover, further in-depth anthropological analysis may help to validate and explain cultural differences in plant use knowledge. For example, by assessing the knowledge of particular caste groups (e.g., Sanyasi), or considering the influence of other factors such as income, landholding size, etc. on biodiversity, ecosystem services, or services to ecosystems at landscape and regional scales.

### Implications for adaptive planning, policy and management

Our evidence indicates a high prevalence of autochthonous, low-cost, small-scale ethnobotanical practices employed to enhance social-ecological resilience to global environmental change. Mobilizing and harnessing this knowledge could build common ground for new partnerships between conservation planners and land managers. Among other co-benefits, cultivating wild and tended plant species can help prevent erosion by erratic and intense rainfall (e.g., *Leucaena leucocephala* (Leucaena)); retain soil moisture during droughts by growing groundcover and enhancing soil fertility (e.g., *Ocimum tenuiflorum* (Sacred basil), *Albizia lebbeck* (Black siris)); serve as windbreaks (e.g., *Dendrocalamus strictus* (Feathery bamboo or various other species of bamboo), *Justicia adhatoda* (Malabar nut)); boost immunity among food insecure communities where malnutrition is high (e.g., *Pueraria tuberosa* (Indian Kudzu)); treat human fever and malaria (e.g., *Centella asiatica* (Indian pennywort), *Crateva unilocularis* (Garlic pear), *Butea monosperma* (Flame of the forest)); treat livestock fever and colds (e.g., *Paris polyphylla* (Herb paris), *Terminalia bellirica* (Yellow myrobalan)); use as pesticides (e.g., *Azadirachta indica* (Neem or Margosa Tree), *Melia azedarach* (Persian lilac)); support alternative livelihoods (e.g., *Bombax ceiba* (Silk cotton tree); or have multiple uses (e.g., *Eupatorium odoratum* (Crofton weed) used for fodder, pest control, medical and religious purposes) [34, 54–56, 58, 59, 105].

Translating best practices from other developing countries could provide useful insights for Nepalese farmers to harness ecosystem service benefits from non-agricultural plants. There is significant potential to reinvest revenue generated from genetic resources in conservation, such as cosmetics, botanical medicines, and pharmaceuticals. Safeguards against Big Pharma expropriation could be strengthened by updating national records of the status and distribution range of non-charismatic protected and threatened species according to IUCN lists (e.g., volumes of Flora of Nepal), or establishing a Traditional Knowledge Digital Library of Nepal, following the example of India [83]. There is a need to up-scale local seed warehouses of useful plants that can withstand dry years, saturation, or invasive populations, as well as increase public spending in purchasing native seeds [88]; and explicitly incorporate the utilization of material on farms in adaptation plans [91]. Tightening legislative controls on access to genetic resources could be supported by becoming a signatory of the Nagoya Protocol on Access to Genetic Resources and Benefit Sharing—a legally binding instrument which aims to monitor, support long-term use, and ensure equitable access for indigenous and future generations [84, 88, 89]. Such local and national initiatives further tie into the ongoing thematic assessment of the sustainable use of wild species of the International Science-Policy Panel on Biodiversity and Ecosystem Services.

## Conclusion

This research provides an original contribution to a growing discourse articulating the role plant biodiversity plays in maintaining ecosystem services and humans’ contributions to biodiversity maintenance. Local knowledge, beliefs, and practices, which have accumulated over generations, support a high diversity of non-agricultural plant communities in the Terai Plains of Nepal. A total of 391 vascular plant specimens belong to 76 distinct plant species from 49 phylogenetic families. This high level of plant agrobiodiversity provides a rich source of ecosystem services that contributes to the social, cultural, environmental, and economic enrichment of Nepalese rice farming communities. Farmers’ knowledge-belief-practice complexes [106], which differ by the caste and age group, assist communities to adapt to emerging risks by enhancing adaptive capacity of both ecosystems and the livelihood resources they provide [73]. However, there appears to be a declining transmission of ethnobotanical knowledge to the younger generation, and farmers associate this trend with declines or loss of formerly tended plants. Results can serve as baseline data to initiate further research, be used to plan for a range of potential development trajectories, and be used to conserve valuable, but disappearing, traditional knowledge and practices.

## Data Availability

Voucher specimens for species were deposited in the National Herbarium and Plant Laboratories in Godawari, Lalitpur in Kathmandu.

## Footnotes

1 Given the domestic origin of plants is difficult to determine, as is what constitutes cultivated or uncultivated, the term ‘tended’ is used to infer a lower level of cultivation of ‘wild species’. For example, farmers may manage the habitat of useful plants that naturally occur, support growth and reproduction through trimming, protect plants from animal, wind or water damage, or monitor harvesting levels.

2 Ensuring we were being sensitive to social norms of disclosure and using reported definitions

## References

[1] Rockström J, Steffen W, Noone K, Persson Å, Chapin FS, Lambin E, Lenton TM, Scheffer M, Folke C, Schellnhuber H, Nykvist B, De Wit CA, Hughes R, van der Leeuw S, Rodhe H, Sörlin S, Snyder PK, Costanza R, Svedin U, Falkenmark M, Karlberg L, Corell RW, Fabry VJ, Hansen J, Walker B, Liverman D, Richardson K, Crutzen P, Foley J (2009). Planetary boundaries: exploring the safe operating space for humanity. Ecol Soc.

[2] Díaz S, Brondizio ES, Ngo HT, Guèze M, Agard J, Arneth A, Balvanera P, Brauman KA, SHM B, KMA C, Garibaldi LA, Ichii K, Liu J, Subramanian SM, Midgley GF, Miloslavich P, Molnár Z, Obura D, Pfaff A, Polasky S, Purvis A, Razzaque J, Reyers B, Chowdhury R, Shin YJ, Visseren-Hamakers IJ, Willis KJ, Zayas CN, IPBES (2019). Summary for policymakers of the global assessment report on biodiversity and ecosystem services of the Intergovernmental Science-Policy Platform on Biodiversity and Ecosystem Services.

[3] Mace GM. Whose conservation? 2014;345(6204):1558–60. 10.1126/science.1254704 25258063

[4] FAO. The state of the world’s biodiversity for food and agriculture. In: Belanger J, Pilling D, editors. FAO commission on genetic resources for food and agriculture assessments. Rome; 2019. p. 572.

[5] Dangi MB, Chaudhary RP (2018). Impacts of environmental change on agroecosystems and livelihoods in Annapurna Conservation Area, Nepal. Environ Dev.

[6] Obersteiner M, Walsh B, Frank S, Havlik P, Cantele M, Liu J, Palazzo A, Herrero M, Lu Y, Mosnier A, Valin H, Riahi K, Kraxner F, Fritz S, van Vuuren D. Assessing the land resource–food price nexus of the Sustainable Development Goals. 2016;2(9):e1501499. 10.1126/sciadv.1501499 PMC5026423 27652336

[7] Hossain MS, Dearing JA, Rahman MM, Salehin M (2016). Recent changes in ecosystem services and human well-being in the Bangladesh coastal zone. Reg Environ Chang.

[8] Daily GC (1997). Nature’s services: Societal dependence on natural ecosystems.

[9] Aleksandrowicz L, Green R, Smith P, Haines A (2016). The impacts of dietary change on Greenhouse gas emissions, land use, water use, and health: a systematic review. PLoS ONE.

[10] Assembly, U.G. Transforming our world: the 2030 Agenda for Sustainable Development, 2015, A/RES/70/1, [cited 2019 25 November]. 2015 available at: https://www.refworld.org/docid/57b6e3e44.html.

[11] Springmann M, Godfray CJ, Rayner M, Scarborough P. Analysis and valuation of the health and climate change cobenefits of dietary change. 2016;113(15):4146–51. 10.1073/pnas.1523119113 PMC4839446 27001851

[12] Foley JA, Ramankutty N, Brauman KA, Cassidy ES, Gerber JS, Johnston M, Mueller ND, O'Connell C, Ray DK, West PC, Balzer C, Bennett EM, Carpenter SR, Hill J, Monfreda C, Polasky S, Rockstrom J, Sheehan J, Siebert S, Tilman D, Zaks DPM (2011). Solutions for a cultivated planet. Nature..

[13] Rosegrant MW, Cline SA (2003). Global food security: challenges and policies. Science..

[14] Bhattarai KR, Ghimire MD (2006). Commercially important medicinal and aromatic plants of Nepal and their distribution pattern and conservation measure along the elevation gradient of the Himalayas. Banko Janakari.

[15] Burlakoti C, Kunwar RM, Jha PK, Karmacharya SB, Chettri MK, Thapa CB, Shrestha BB (2008). Folk herbal medicines of Mahakali Watershed Area, Farwest Nepal. Medicinal Plants in Nepal: an anthology of contemporary research.

[16] Kunwar RM, Mahat L, Acharya RP, Bussman RW (2013). Medicinal plants, traditional medicine, markets and management in far-west Nepal. J Ethnobiol Ethnomed.

[17] Kumar BM, Nair PKR (2004). The enigma of tropical homegardens. Agrofor Syst.

[18] Clarke LW, Li L, Jenerette GD, Yu Z (2014). Drivers of plant biodiversity and ecosystem service production in home gardens across the Beijing Municipality of China. Urban Ecosyst.

[19] Lubbe CS, Siebert SJ, Cilliers SS (2011). Floristic analysis of domestic gardens in the Tlokwe City Municipality, South Africa. Bothalia.

[20] International Institute for Environment and Development. Smallholder innovation for resilience: promoting resilient farming systems and local economies. 2015; [cited 2019 25 November] [Available from: http://www.biocultural.iied.org/smallholder-innovation-resilience-sifor.

[21] Seto KC, Güneralp B, Hutyra LR (2012). Global forecasts of urban expansion to 2030 and direct impacts on biodiversity and carbon pools. Proc Natl Acad Sci.

[22] Haenke S, Kovacs-Hostyanszki A, Freund J, Batary P, Jauker B, Tscharntke T, Holzschuh A (2014). Landscape configuration of crops and hedgerows drives local syrphid fly abundance. J Appl Ecol.

[23] Ford A, Nigh R (2015). The Maya forest garden: Eight millennia of sustainable cultivation of the tropical woodlands.

[24] Grieg-Gran M, Gemmill-Herren B (2011). Handbook for participatory socio-economic evaluation of pollinator-friendly practices.

[25] Elmqvist T, Folke C, Nystrom M, Peterson G, Bengtsson J, Walker B, Norberg J (2003). Response diversity, ecosystem change, and resilience. Front Ecol Environ.

[26] Fahrig L (1997). Relative effects of habitat loss and fragmentation on population extinction. J Wildl Manag.

[27] Acharya KP (2006). Linking trees on farms with biodiversity conservation in subsistence farming systems in Nepal. Biodivers Conserv.

[28] Thorn JPR, Thornton TF, Helfgott A (2015). Autonomous adaptation to global environmental change in peri-urban settlements: Evidence of a growing culture of innovation and revitalisation in Mathare Valley Slums, Nairobi. Glob Environ Chang.

[29] Thorn JPR. Adaptation “from below” to changes in species distribution, habitat and climate in agro-ecosystems in the Terai Plains of Nepal. Ambio. 2019:1–16. 10.1007/s13280-019-01202-0 PMC6882764 31183689

[30] Lattice SD (2008). Multivariate data visualization.

[31] United States Department of Agricultural Resources Natural Resources Conservation Science. Plants database. 2015; [cited 2019 25 November 2019 ]; Available from: https://www.nrcs.usda.gov/wps/portal/nrcs/site/national/home/.

[32] Ehrlich PR, Ehrlich AH (2014). Can a collapse of global civilization be avoided?. Proc R Soc B.

[33] Mohri H, Lahoti S, Saito O, Mahalingam A, Gunatilleke N, Irham, Hoang VT, Hitinayake G, Takeuchi K, Herath S (2013). Assessment of ecosystem services in homegarden systems in Indonesia, Sri Lanka, and Vietnam. Ecosys Serv.

[34] Bhattarai S, Chaudhary RP, Taylor RS (2006). Ethnomedicinal plants used by the people of Manang district, central Nepal. J Ethnobiol Ethnomed.

[35] Kunwar RM, Shrestha KP, Bussmann RW (2010). Traditional herbal medicine in Far-west Nepal: a pharmacological appraisal. J Ethnobiol Ethnomed.

[36] Manandhar NP (1998). Native phytotherapy among the Raute tribe of Dadeldhura district, Far-west Nepal. J Ethnopharmacol.

[37] Garibaldi A, Turner N (2004). Cultural keystone species: implications for ecological conservation and restoration. Ecol Soc.

[38] Guneratne A (2002). Many tongues, one people: the making of Tharu identity in Nepal.

[39] Paudel PK, Bhattarai BP, Kindlmann P, Kindlmann P (2011). An overview of the biodiversity in Nepal. Himalayan biodiversity in the changing world.

[40] Guo Z, Zhang L, Li Y (2010). Increased dependence of humans on ecosystem services and biodiversity. PLoS ONE.

[41] World Bank. In: World Bank, editor. Climate risk and adaptation country profiles. USA; 2015.

[42] World Food Programme, World Food Programme (2010). Country Overview Nepal.

[43] Luitel DR, Rokaya MB, Timsina B, Münzbergová Z (2014). Medicinal plants used by the Tamang community in the Makawanpur district of central Nepal. J Ethnobiol Ethnomed.

[44] Government of Nepal. Nepal Census of Agriculture 2001/2. 2002 [cited 2014 26 December ]; Available from: https://cbs.gov.np.

[45] Regmi RR (1994). Deforestation and rural society in the Nepalese Terai. Occas Pap Sociol Anthropol.

[46] Bridson DM, Forman L (1998). The herbarium handbook.

[47] Press JR, Shrestha KK, Sutton DA (2000). Annotated checklist of the flowering plants of Nepal.

[48] Bernard HR, Gravlee CC (2014). Handbook of methods in cultural anthropology.

[49] Brush SB, Brush SB, Stabinsky D (1996). Is common heritage outmoded?. Valuing local knoweldge: indigenous people and intellectual property rights.

[50] Olango TM, Tesfaye B, Catellani M, Pe ME (2014). Indigenous knowledge, use and on-farm management of enset (Ensete ventricosum (Welw.) Cheesman) diversity in Wolaita, Southern Ethiopia. J Ethnobiol Ethnomed.

[51] Shannon C, Weaver W (1949). The mathematical thoery of communication.

[52] R Development Core Team (2005). R: A Language and environment for statistical computing.

[53] Clarke KR, Gorley RN. PRIMER v6: User Manual/Tutorial. United Kingdom; 2006. p. 192.

[54] Storrs A, Storrs J (1998). Trees and shrubs of Nepal and the Himalayas.

[55] IUCN Nepal (2004). National register of medicinal and aromatic plants (Revised and updated).

[56] Singh AG, Kumar A, Tewari DD (2012). An ethnobotanical survey of medicinal plants used in Terai forest of western Nepal. J Ethnobiol Ethnomed.

[57] Pigg SL (1992). Inventing social categories through place: Social representations and development in Nepal. Comp Stud Soc Hist.

[58] Mishra B. Scientific name agro-forestry crops of Nepal. 2003 [cited 2015 25 March 2015]; Available from: https://bijeshmishra.wordpress.com/2012/04/01/scientific-name-agro-forestry-crops-of-nepal/.

[59] Government of Nepal (2014). Plant resources (a scientific publication).

[60] Kerkhoff EE (2003). Sustainable sloping lands and watershed management conference.

[61] Mandal D, Panda AK, Rana M (2013). Medicinal plants used in folk medicinal practice available in rich biodiversity of Sikkim. Environ Ecol.

[62] World Flora Online (WFO). World Flora Online. Available online: http://www.worldfloraonline.org. Accessed on 22 March 2020; 2020.

[63] Dangol DR (2008). Traditional uses of plants of commonland habitats in Western Chitwan, Nepal. J Inst Agric Anim Sci.

[64] Chatterjee K, Ali KM, De D, Panda DK, Ghosh D (2012). Anti diabetic and anti oxidative potencies study of ethyl acetate fraction of hydromethanolic (40:60) extract of seed of Eugenia jambolana Linn and its chromatographic purification. J Pharm Res.

[65] Kew Royal Botanic Gardens. World checklist of selected plant families. Facilitated by the Royal Botanic Gardens, Kew. 2015; [cited 10 October 2015]; Available from: http://apps.kew.org/wcsp/.

[66] Subedi BP, Chintamani LD, Messeschmidt DA (1998). Tree and land tenure in the Eastern Terai, Nepal: A case study from the Siraha and Saptari districts, Nepal.

[67] Kunwar RM, Bussmann RW (2006). *Ficus* (Fig) species in Nepal: a review of diversity and indigenous uses. Iyonia.

[68] Chisholm H (1911). Encyclopædia Britannica 4. Vol.

[69] Pattanayak BP, Das D, Panda SK (2010). *Ocimum sanctum Linn.* A reservoir plant for therapeutic applications: an overview. Pharmacogn Rev.

[70] Dangol DR, Maharjan KL, Maharjan SK, Acharya AK, Joshi BK, HB KC, Acharya AK (2017). Wild edible plants of Nepal. Conservation and Utilization of Agricultural Plant Genetic Resources in Nepal.

[71] Kunwar RM, Duwadee NPS (2003). Ethnobotanical notes on flora of Khaptad national park, far western Nepal. Himal J Sci.

[72] UK Government Office for Science (2011). Foresight. The future of food and farming: Challenges and choices for global sustainability (Executive summary).

[73] Government of Nepal Ministry of Science Technology and Environment (2015). Indigenous and local knowledge and practices for climate resilience in Nepal, mainstreaming climate change risk management in development.

[74] Ingold T (2000). Perception of the environment: essays on livelihood, dwelling and skill.

[75] Gurung B (1994). A cultural approach to natural resource management: a case study from Eastern Nepal. Summary report of FAO regional expert consultation on non-wood forest products: social, economic and cultural dimensions.

[76] Del Angel-Perez AL, Mendoza MA (2004). Totonac homegardens and natural resources in Veracruz, Mexico. Agric Human Values.

[77] Salas M (2005). Seed songs—reflections on swidden agriculture agrobiodiversity and food sovereignty. Indigenous Affairs.

[78] UNEP (2006). Guidelines for the rapid assessment of inland, coastal and marine wetland biodiversity, in 9th Meeting of the Conference of the Parties to the Convention on Wetlands (Ramsar, Iran, 1971): Wetlands and water: supporting life, sustaining livelihoods.

[79] Boelee E, Chiramba T, Khaka E (2011). An ecosystem services approach to water and food security in An ecosystem services approach to water and food security, International Water Management Institute and United Nations Environment Programme.

[80] Kunwar R, Thapa K, Shrestha R, Shrestha P, Bhattarai N, Tiwari N, Shrestha K (2013). Medicinal and Aromatic Plants Network (MAPs-Net) Nepal: An open access digital database. Banko Janakari.

[81] Bhagwat SA, Breman E, Thekaekara T, Thornton TF, Willis KJ (2012). A Battle Lost? Report on two centuries of invasion and management of *Lantana camara L.* in Australia, India and South Africa. PLoS ONE.

[82] Ango TG, Börjeson L, Senbeta F, Hylander K (2014). Balancing ecosystem services and disservices: smallholder farmers’ use and management of forest and trees in an agricultural landscape in southwestern Ethiopia. Ecol Soc.

[83] Comberti C, Thornton TF, Wyllie de Echeverria V, Patterson T (2015). Ecosystem services or services to ecosystems? Valuing cultivation and reciprocal relationships between humans and ecosystems. Glob Environ Chang.

[84] Berkes F (1999). Sacred ecology: traditional ecological knowledge and resource management.

[85] Hunn ES (2002). Evidence for the precocious acquisition of plant knowledge by Zapotec children. Ethnobiology Biocultural Diversity.

[86] Pfeiffer JM, Butz RJ (2005). Assessing cultural and ecological variation in ethnobiological research: the importance of gender. J Ethnobiol.

[87] Saslis-Lagoudakis CH, Hawkins JA, Greenhill SJ, Pendry CA, Watson MF, Tuladhar-Douglas W, Baral SR, Savolainen V (2014). The evolution of traditional knowledge: environment shapes medicinal plant use in Nepal. Proc R Soc B.

[88] World Resources Institute (2005). World Resources - 2005. The wealth of the poor: managing ecosystems to fight poverty.

[89] Poudel S, Kotani K (2012). Climatic impacts on crop yield and its variability in Nepal: do they vary across seasons and altitudes?. Clim Chang.

[90] Ghimire SK, Jha PK, Karmacharya SB, Chettri MK, Thapa CB, Shrestha BB (2008). Medicinal plants in the Nepal Himalaya: current issues, sustainable harvesting, knowledge gaps and research priorities. Medicinal Plants in Nepal: an anthology of contemporary research.

[91] Kunwar RM, Nepal BK, Kshetri HB, Rai SK, Bussmann RW (2006). Ethnomedicine in Himalaya: a case study from Dolpa, Humla, Jumla and Mustang districts of Nepal. J Ethnobiol Ethnomed.

[92] Manandhar NP. Role of ethnobotany in the context of Nepal; Paper presented on National Training Workshop on "Application of ethnobotany to community development" held in Sauraha, Chitwan from January 6–13, 1997; 1997.

[93] Zhang L, Chai Z, Zhang Z, Geng Y, Wang Y (2016). 2016. Ethnobotanical study of traditional edible plants used by the Naxi people during drought. J Ethnobiol Ethnomed.

[94] Balemie K, Kebebew F (2006). Ethnobotanical study of wild edible plants in Derashe and Kucha Districts, South Ethiopia. J Ethnobiol Ethnomed.

[95] Government of India. Traditional knowledge digital library. 2016 [cited 2016; Available from: http://www.tkdl.res.in/tkdl/langdefault/common/Home.asp?GL=Eng.

[96] Royal Botanical Gardens Kew (2016). The state of the world’s plants report – 2016.

[97] Nair KPP (2013). The agronomy and economy of turmeric and ginger: the invaluable medicinal spice crops.

[98] Wynberg R, van Niekerk J (2014). Global ambitions and local realities: achieving equity and sustainability in two high-value natural product trade chains. Forests Trees Livelihoods.

[99] Twilley N (2015). Who owns the patent on nutmeg?. The New Yorker.

[100] Convention of Biological Diversity. Nepal overview. 2016 [cited 2016 12 May ]; Available from: https://www.cbd.int/countries/profile/default.shtml?country=np#measures.

[101] Prip C, Rosendal K (2015). Access to genetic resources and benefit-sharing from their use (ABS)—state of implementation and research gaps.

[102] Madhav KC, Phoboo S, Jha PK (2010). Ecological study of *Paris Polyphylla Sm*. Ecoprint.

[103] Kunwar RM, Nepal BK, Kshhetri HB, Rai SK, Bussman RW (2006). Ethnomedicine in Himalaya: a case study from Dolpa, Humla, Jumla and Mustang districts of Nepal. J Ethnobiol Ethnomed.

[104] Kunwar RM, Adhikari N (2005). Ethnomedicine of Dolpa district, Nepal: the plants, their vernacular names and uses. Lyonia..

[105] Paudyal A, Regmi B, Bordoni P (2009). Climate change and agrobiodiversity in Nepal: Opportunities to include agrobiodiversity maintenance to support Nepal’s National Adaptation Programme of Action (NAPA).

[106] Berkes F (1999). Sacred ecology: Traditional ecological knowledge and management systems.

